# Naringenin restores neuronal membrane electro-biophysical homeostasis: insights from EIS circuit modeling and molecular dynamics

**DOI:** 10.3389/fmed.2026.1861895

**Published:** 2026-08-31

**Authors:** Yiyao Zhang, Cai You, Shimeng Sun, Tian Fang, Qing Ma, Shangwei Huang, Yi Ding, Han Li, Yiyong Chen, Jianfei Zhang, Jia Xu

**Affiliations:** 1 Department of Physiology and Pharmacology, NBU Health Science Center, Ningbo University, Ningbo, China; 2 Department of Clinical Laboratory, The Affiliated Yangming Hospital of Ningbo University, Yuyao China; 3 Department of Neurological Surgery, The First Affiliated Hospital of Ningbo University, Ningbo, China

**Keywords:** CPE-equivalent circuit model, electrical impedance spectrum, molecular simulation, naringenin, oxidative stress

## Abstract

**Introduction:**

Naringenin (NGN), a bioactive flavonoid derived from Citri Reticulatae Pericarpium, exhibits considerable neuroprotective properties. However, the precise mechanisms underlying its restoration of neuronal membranes during oxidative stress remain elusive.

**Methods:**

This study utilized an integrated multiscale approach combining network pharmacology, electrical impedance spectroscopy (EIS) with an eight-element equivalent circuit model, biochemical assays, and molecular dynamics (MD) simulations to systematically evaluate NGN's effects on H2O2-induced injury in HT22 neurons.

**Results:**

EIS analysis revealed that NGN significantly restored membrane biophysical integrity, reflected by the recovery of critical electro-biophysical parameters (Rm, Cm and fc1) at subcellular resolution. Concurrently, biochemical analyses showed that NGN activates the NRF2/HO-1 signaling pathway, resulting in elevated superoxide dismutase (SOD) activity and decreased malondialdehyde (MDA) levels, thereby enhancing endogenous antioxidant defenses. Furthermore, molecular docking and MD simulations confirmed at the atomic level that NGN forms highly stable, high-affinity complexes with core antioxidant and anti-apoptotic targets, including NRF2, HO-1, and BCL-2.

**Discussion:**

These findings collectively indicate that NGN exerts a dual neuroprotective mechanism involving both biochemical antioxidant defense and biophysical membrane restoration. Integrating traditional pharmacology with modern biophysical modeling, this study positions EIS as an innovative, label-free approach for monitoring membrane protection, thereby establishing an electrophysiology-based platform for both mechanistic investigation and clinical translation of flavonoids in neuroprotection.

## 1 Introduction

Oxidative stress constitutes a central pathological mechanism in neuronal injury and death, playing a pivotal role in the progression of numerous neurodegenerative disorders (1). This pathology fundamentally arises from an imbalance between the production and clearance of reactive oxygen species (ROS), triggering destructive cascading reactions such as lipid peroxidation and protein oxidation (2). Ultimately, this oxidative onslaught compromises cell membrane integrity, disrupts ion homeostasis, and instigates organelle dysfunction (3, 4). Neurons are particularly susceptible to such damage due to their high metabolic demand, membranes rich in polyunsaturated fatty acids, and relatively restricted endogenous antioxidant defenses. Consequently, *in vitro* models employing hydrogen peroxide (H_2_O_2_) as a stable ROS source have become a gold standard for evaluating neuroprotective compounds (5, 6). Indeed, this model has been instrumental in elucidating the antioxidant efficacies of diverse agents—including N-acetylcysteine (7), polyphenols such as resveratrol (8), and synthetic drugs like epalrestat (9)—through mechanisms ranging from MAPK modulation to AKT/CREB activation. Building on these established paradigms, identifying novel, multi-targeted active substances capable of antagonizing ROS-associated neuronal damage remains a critical research imperative.

*Citri Reticulatae Pericarpium* (CRP), commonly known as aged tangerine peel, is a highly regarded traditional Chinese medicine (TCM) derived from the Rutaceae family, epitomizing the philosophy of “homology of medicine and food” (10, 11). CRP is exceptionally rich in bioactive flavonoids, establishing it as a potent natural antioxidant source (12, 13). Among its constituents, naringenin (NGN)—a well-characterized dihydroflavonoid—has exhibited remarkable neuroprotective potential. Classical biochemical studies demonstrate that NGN effectively mitigates lipid peroxidation (indicated by reduced malondialdehyde, MDA) and revitalizes endogenous antioxidant enzymes such as superoxide dismutase (SOD) (14, 15), largely through the systematic activation of the NRF2/HO-1 cellular defense pathway (16).

Despite this well-documented biochemical efficacy, a critical spatio-temporal knowledge gap remains regarding NGN's precise neuroprotective sequence. Recognizing that oxidative injury is a multi-stage process, the initial physical disruption of the cell membrane occurs significantly earlier than the orchestration of protective genomic responses. The neuronal cell membrane is not merely a physical boundary; it is the fundamental basis for electrophysiological function and ion homeostasis, making it the primary frontier attacked by ROS (17–19). H_2_O_2_-induced lipid peroxidation rapidly compromises this membrane integrity, often triggering irreversible cellular dysfunction before downstream intracellular genomic responses (such as NRF2 activation) can fully mount a defense (20–23). Traditionally, neuronal membrane damage is evaluated using biochemical endpoint assays (e.g., lactate dehydrogenase leakage) (24) or fluorescent lipid probes (25). However, these conventional methods are largely destructive, label-dependent, and incapable of capturing continuous, subcellular biophysical dynamics. As a result, sensitive, label-free biophysical evidence remains scarce to determine whether NGN can directly and rapidly stabilize neuronal membranes against early-onset oxidative damage at the electro-biophysical level. Therefore, investigating NGN's protective efficacy from this novel biophysical perspective—and integrating it with its well-established biochemical pathways—is essential for a comprehensive understanding of its multidimensional mechanisms.

To bridge this macroscopic physical and microscopic molecular divide, a multi-scale analytical strategy is required. Electrical Impedance Spectroscopy (EIS) provides a sensitive, non-invasive, and label-free platform to quantitatively evaluate cellular electro-biophysical properties via complex impedance (Z). EIS enables the real-time detection of membrane ultrastructural alterations, permeability shifts, and physiological states at a subcellular resolution (26–30). Concurrently, to anchor these macroscopic biophysical observations to specific molecular targets, network pharmacology offers a systemic framework for mapping component-target-disease interactions (31, 32). Furthermore, Molecular Dynamics (MD) simulations can be subsequently employed to validate these network predictions, providing kinetic, atomic-level evidence of the drug's binding stability with core targets (33). Thus, integrating these biophysical and computational methodologies establishes a robust multidisciplinary framework. This combined approach enables the systematic correlation of macroscopic electro-biophysical membrane responses with specific drug-target interactions and their downstream signaling pathways.

Therefore, this study systematically investigates the neuroprotective effects of NGN against H_2_O_2_-induced neuronal injury through an innovative, multi-dimensional framework. By integrating EIS-based equivalent circuit modeling with network pharmacology, biochemical assays, and molecular dynamics simulations, we aim to elucidate a synergistic dual mechanism: the biophysical restoration of neuronal membrane integrity and the activation of antioxidant signaling pathways. Ultimately, this work provides novel mechanistic insights into NGN and establishes a pioneering paradigm for evaluating membrane-targeting natural compounds in neurodegeneration.

## 2 Materials and methods

### 2.1 Chemicals and reagents

The HT22 mouse hippocampal neuronal cell line was obtained from Fuheng Biotechnology Co., Ltd. (Shanghai, China). NGN (≥98.0%) was sourced from Solarbio Science & Technology Co., Ltd. (Beijing, China) and dissolved in 100 mmol/L DMSO (Macklin, Shanghai, China). The SOD and MDA assay kits were procured from Nanjing Jiancheng Institute of Bioengineering (Nanjing, China) and Grace Biotechnology (Suzhou, China), respectively. The BCA Protein Assay Kit was obtained from Thermo Fisher Scientific Inc. (Waltham, USA). Primary antibodies against HO-1 (66743-1-Ig) and NRF2 (16396-1-AP) were from Proteintech Group (Wuhan, China), and the β-actin antibody (sc-47778) was from Santa Cruz Biotechnology (Dallas, USA). H_2_O_2_ (30%) was supplied by Sinopharm Chemical Reagent Co., Ltd. (Shanghai, China). All other chemicals were acquired from local commercial sources.

### 2.2 Network pharmacology analysis

#### 2.2.1 Compound activity evaluation

To evaluate the compound's activity, its human intestinal absorption rate (HIA) and blood-brain barrier (BBB) permeability of the compound were predicted using the SwissADME platform (http://www.swissadme.ch/index.php) (34). HIA was then classified into three levels: high absorption rate (≥80%), moderate absorption rate (20–80%), and low absorption rate (< 20%).

#### 2.2.2 Compound target prediction and risk target screening

Putative target proteins were fished and filtered conditionally from SwissTargetPrediction (http://www.swisstargetprediction.ch/) (35) and Super-PRED (https://prediction.charite.de/subpages/target_prediction.php) (36). Protein targets associated with antioxidants were identified through the GeneCards database (http://www.genecards.org/) (37), the keywords for the searches were “Antioxidant.” Antioxidants-associated proteins and putative target proteins of candidate compounds were verified using their unique UniProtKB ID and target names in the UniProt database (http://www.uniprot.org/).

#### 2.2.3 Protein network construction, PPI analysis, and functional enrichment

To construct the protein-protein interaction (PPI) network, experimentally validated interactions for the putative proteins were obtained from the STRING database (version 12.0) and imported into Cytoscape software (version 3.10.2) (http://www.cytoscape.org/) (38). Highly interconnected protein subnetworks (clusters) were detected using the MCODE plugin. Hub proteins (key nodes) were identified based on key topological parameters of the PPI network, including degree, betweenness centrality, and closeness centrality. Kyoto Encyclopedia of Genes and Genomes (KEGG) pathway enrichment analyses and Gene Ontology (GO) for the putative proteins or significant subnetworks were conducted using DAVID (https://davidbioinformatics.nih.gov/). Putative proteins or modules with an enrichment *p-value* ≤ 0.01 were considered statistically significant. The enrichment results were presented as bar charts and bubble plots generated with ImageGP (https://www.bioinformatics.com.cn/) to elucidate the potential antioxidant mechanisms of NGN at the network pharmacology level. Compound-target-pathway networks were also built and visualized using Cytoscape.

### 2.3 Cell culture and viability assay

HT22 cells were maintained in high-glucose Dulbecco's Modified Eagle Medium (DMEM) containing 10% fetal bovine serum (FBS) and 1% penicillin-streptomycin (100 U/mL penicillin, 100 μg/mL streptomycin) at 37°C in a 5% CO_2_ atmosphere. A stock solution of NGN was prepared by dissolution in DMSO at 100 mmol/L, followed by dilution in culture medium to a final DMSO concentration below 0.1%. For cytotoxicity assessment, HT22 cells were plated in 96-well plates at 3.5 × 10^4^ cells/mL and allowed to adhere overnight. Cells were then exposed to H_2_O_2_ (50–1,000 μmol/L) or NGN (10–100 μmol/L, prepared in serum-free DMEM) for 24 h. To evaluate NGN's protective effects, HT22 cells at 70% confluence were pretreated with 200 μmol/L H_2_O_2_ for 4 h, followed by NGN treatment for 20 h. Cell viability was determined using the MTT assay, with absorbance measured at 490 nm on a Varioskan LUX microplate reader (Thermo Fisher Scientific, USA).

### 2.4 Cellular impedance spectroscopy analysis

#### 2.4.1 EIS measurements

Trypsin was used to harvest treated HT22 cells. The desired cell volume fraction was diluted for AC electrical impedance measurements. DMEM medium without FBS and penicillin/streptomycin (P/S) was used as the cell detection buffer. EIS measurements of the HT22 cell suspension were obtained using an Agilent 4294A impedance analyzer (Agilent Technologies, Japan) equipped with a 42942A adapter and 16192A fixture. Each sample was measured from 10 kHz to 100 MHz with 124 frequency points at room temperature. The impedance parameters of DMEM were obtained to standardize data processing. Standardization and data processing followed the previously described methods (39). Briefly, cell impedance (*Z* = *Z*_*c, cell*_) was determined as the difference between the measured impedance (*Z*_*m, measure*_) and extracellular fluid impedance (*Z*_*medium*_ = *Z*_*p, polarization*_). Complex impedance was expressed as $Z_{m}=\left|Z_{m}\right|\times e^{-j\theta }={Z^{\prime }}_{m}+j{Z^{\prime \prime }}_{m}$. Real $\left({Z^{\prime }}_{m}\right)$ and imaginary $\left({Z^{\prime \prime }}_{m}\right)$ impedances were calculated as ${Z^{\prime }}_{m}=\left|Z_{m}\right|\times \cos\theta$ and ${Z^{\prime \prime }}_{m}=\left|Z_{m}\right|\times \sin\theta$, respectively. For each concentration point, measurements were performed in triplicate, and the mean value was taken as the representative measurement for that concentration. Across the entire concentration range, the mean values of all concentration points are presented as mean ± SD. The same procedure was applied to both the measurement and data fitting of the EIS impedance data.

#### 2.4.2 Cole–Cole mathematical model parameter analysis

HT22 cells have a nucleus, and there are two β-dispersions at the interface of the cell membrane and nuclear membrane. We define the general 9-parameter dual-dispersion Cole–Cole model of impedance spectroscopy as follows Equation 1:


$$\begin{array}{l} Z\left(s\right)=R_{\infty }+\frac{\Delta R_{1}}{1+{\left(jf/f_{c1}\right)}^{\beta_{1}}}+\frac{\Delta R_{2}}{1+{\left(jf/f_{c2}\right)}^{\beta_{2}}} \end{array}$$ (1)

Where *R*_∞_ is the extra high-frequency resistance to accommodate the non-zero value of the impedance at high frequencies. Δ*R*_1_, Δ*R*_2_, *f*, *f*_*C*1_, *f*_*C*2_, β_1_, and β_2_ represent the low- and high-frequency relaxation increments, frequency, characteristic frequencies, and Cole–Cole parameters (0 < β < 1), respectively (40), *j* is the imaginary unit ($j=\sqrt{-1}$). Res(*Z*′) and Res(*Z*″) were calculated using the Cole–Cole mathematical model from the experimental real (*Z*′) and imaginary (*Z*″) impedance and the theoretical real $\left({Z^{\prime }}_{theory}\right)$ and imaginary $\left({Z^{\prime \prime }}_{theory}\right)$ impedance using the following Equation 2 to minimize the residual:


$$\begin{array}{l} Res(Z^{\prime })=\frac{\sum \left|Z^{\prime }-{Z^{\prime }}_{theory}\right|}{\Delta Z^{\prime }}\times \frac{100}{n},\\ \;Res(Z^{\prime \prime })=\frac{\sum \left|Z^{\prime \prime }-{Z^{\prime \prime }}_{theory}\right|}{\Delta Z^{\prime \prime }}\times \frac{100}{n}, \end{array}$$ (2)

Where Δ is the difference between the maximum and minimum values of the relevant observed values within the considered frequency range, and *n* is the number of frequency points covered by the frequency range (41).

#### 2.4.3 Neuronal equivalent circuit model construction and parameter analysis

Mouse hippocampal neurons may not be an ideal capacitor as assumed by standard electrical elements (42). Thus, to better simulate the spectral characteristics of cellular electrical impedance, an eight-element equivalent circuit model was optimally designed for this study according to the sub-microscopic structural characteristics of neurons (39). The constant phase element (CPE) model provided the best fit in terms of accuracy for the experimental data (43). This model applies to the state of the cell suspension under the assumed condition that the structural electrical characteristics of all cells in the sample being tested are the same. A suspension consisting of several identical cells (the sample to be tested) can reflect the average state of identical individual cells.

**Figure 1:**
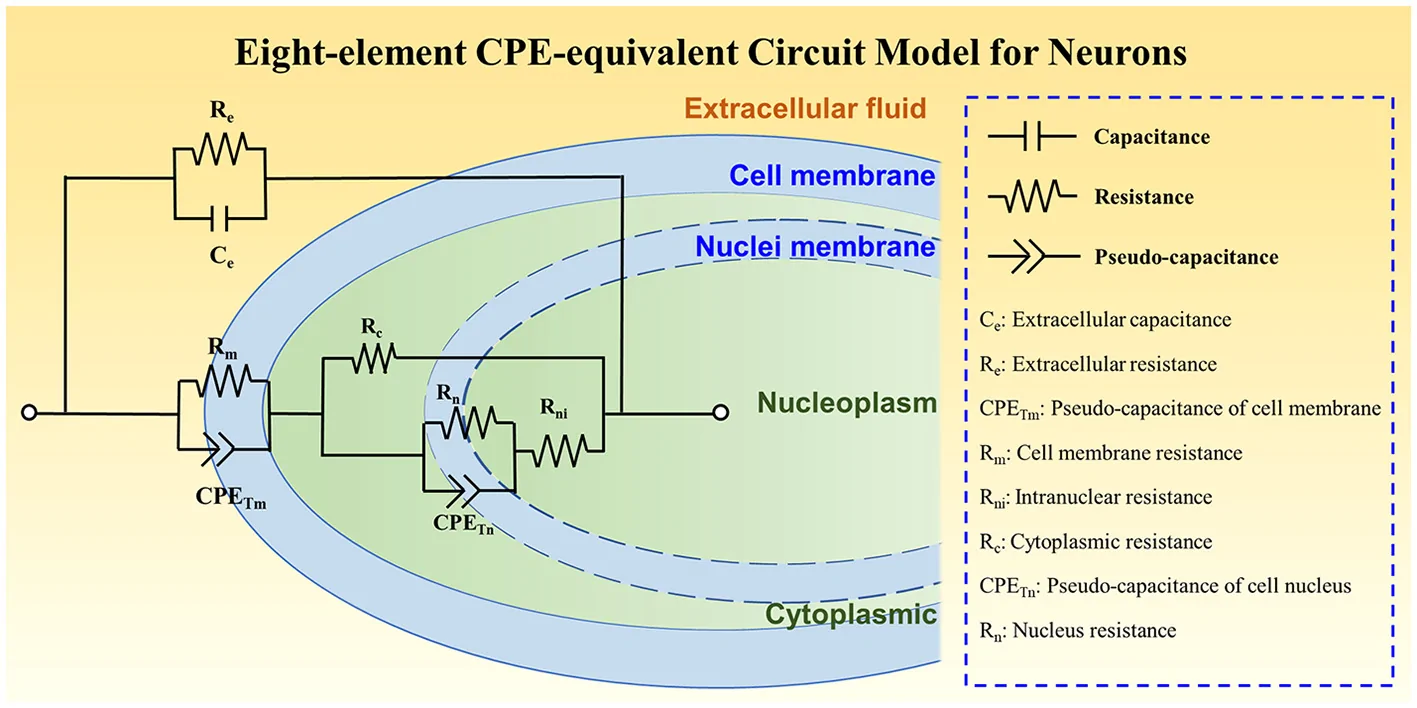
Schematic diagram of the eight-element constant phase element (CPE)-equivalent circuit model for neuronal electrical impedance analysis. The extracellular fluid is modeled by a series combination of *R*_*e*_ (extracellular resistance) and *C*_*e*_ (extracellular capacitance), representing its conductive and capacitive properties. The neuronal membrane system includes R_m_ (membrane resistance) in parallel with *CPE*_*Tm*_ (Pseudo-capacitance of cell membrane, a membrane constant phase element accounting for frequency-dependent capacitive behavior). The nuclear compartment consists of *R*_*n*_ (nucleus resistance, nuclear membrane resistance) in parallel with *CPE*_*Tn*_ (Pseudo-capacitance of cell nucleus, a nuclear membrane constant phase element). Intracellular regions are purely resistive: *R*_*ni*_ (intranuclear resistance) and *R*_*c*_ (cytoplasmic resistance) are connected in series within the nuclear and cytoplasmic spaces, respectively. Total impedance is derived from the hierarchical series-parallel integration of these elements, enabling quantitative analysis of frequency-dependent neuronal impedance responses.

In this model (Figure 1), the cell membrane and nuclear membrane have their own resistance (*R*_*m*_ and *R*_*n*_, respectively) and pseudo-capacitance (*CPE*_*Tm*_, *CPE*_*Tn*_), while the intranuclear and cytoplasmic are purely resistive (*R*_*ni*_, *R*_*c*_). The equivalent parallel resistance (*R*_*e*_) and capacitance (*C*_*e*_) circuit represent the capacitance of the extracellular fluid. The impedance in terms of the parallel/serial combinations of elements of the eight-element CPE-equivalent circuit model is given by Equation 3


$$\begin{array}{l} Z=\;\left(C_{e}∥Z_{e}\right)∥\left\{\left\{\;\left[\left(Z_{CPE_{n}}∥Z_{n}\right)+Z_{ni}\right]∥Z_{c}\right\}+\;\left(Z_{CPE_{m}}∥Z_{m}\right)\right\} \end{array}$$ (3)

Where *Z*_*k*_ = *R*_*k*_, for κ ∈ (*e, m, n, ni, c*), $Z_{k}\left(s\right)={\left(S^{\alpha_{l}}\cdot CPE\text{\_}T_{l}\right)}^{-1}$ for *l* ∈ (*m, n*) while 0 < CPE_T < 1 and α = *CPE*_*p*_.

As a result, the impedance of the equivalent circuit model using fractional circuit theory (44) becomes


$$\begin{array}{l} Z\left(s\right)=\{\{{\left\{{\left[\frac{1}{\left(S^{\alpha_{n}}\right)CPE_{Tn}+\frac{1}{R_{n}}}+\frac{1}{R_{ni}}\right]}^{-1}+\frac{1}{R_{c}}\right\}}^{-1}\\ \;{{+\frac{1}{\left(S^{\alpha_{m}}\right)CPE_{Tm}+\frac{1}{R_{m}}}\}}^{-1}+{\left(Ce+\frac{1}{R_{e}}\right)}^{-1}\}}^{-1} \end{array}$$ (4)

Where *s* = *jω*. The complex impedance corresponding to Equation 4 is evaluated using the substitution $s^{\alpha_{i}}=\omega^{\alpha_{i}}\left[\cos\left(\frac{\pi \cdot \alpha_{i}}{2}\right)+j\sin\left(\frac{\pi \cdot \alpha_{i}}{2}\right)\right]$. All parameters were derived by automated curve fitting of impedance spectra obtained from HT22 neuronal cells, performed with Zview software (v3.1, Scribner Associates Inc., Southern Pines, NC, USA).

### 2.5 Biochemical verification and quantitative analysis

#### 2.5.1 Determination of SOD activity and MDA level

After collecting HT22 cells and discarding the supernatant, add extraction solution and subject to ice-bath sonication. Centrifuge at 12,000 rpm for 10 min. The supernatants were then collected for measurement of SOD activity and MDA level, according to the manufacturer's instructions. SOD activity was measured using the xanthine oxidase method, while MDA level was determined using the thiobarbituric acid (TBA) colourimetric method.

#### 2.5.2 Western blot analysis

Total protein samples were extracted from each group of HT22 cells and quantified using the BCA assay kit. Protein samples were electrophoresed on 8–12% SDS-PAGE gels at 80 V for 40 min followed by 120 V for 60 min. Subsequently, separated proteins were transferred to PVDF membranes (Millipore, Billerica, MA, USA) at 300 mA for 1 h. After blocking with 5% dried skim milk for 2 h at room temperature, the membranes were incubated with primary antibodies against HO-1 (1:1000), NRF2 (1:1000) and β-actin (1:1000) at 4 °C overnight. After washing six times (5 min each time) with TBST solution, the membranes were incubated with secondary antibodies at room temperature for 1 h. The membranes were then washed three more times with TBST solution (10 min each time). Protein expression was determined by enhanced chemiluminescence (ECL, NCM Biotech, China) using an automated luminescence imaging system (Tanon-5300 Multi, China). The relative expression levels of target proteins were evaluated using ImageJ software and normalized to that of β-actin.

### 2.6 Molecular docking

Molecular docking was performed using AutoDockTools-1.5.6 to investigate binding interactions between NGN (CID: 439246) and target proteins (NRF2: 2LZ1; HO-1: 1N45; SRC: 1YOJ; ESR1: 2OUZ; PPARG: 8WFE; GSK3β: 1I09; BCL-2: 5WHI) following an established protocol (45) with modifications. The 3D structures of NGN and target proteins were retrieved from PubChem and the Protein Data Bank (http://www.pdb.org/), respectively. Protein structures were prepared by removing water molecules and adding polar hydrogens with assigned charges. AutoDock Vina was used to determine the minimum binding energy, while PyMOLWin and DiscoveryStudio45 were employed to visualize and analyze the binding sites and interaction patterns.

### 2.7 Molecular dynamics simulation

Molecular dynamics simulations were conducted for 100 ns using GROMACS-2025.1 with the CHARMM27 all-atom force field and TIP3P water model (46). The protein structure was prepared using Swiss-PDB Viewer (47), while the ligand parameters were generated via Sobtop and Multiwfn (48). The complex was solvated in a dodecahedron box (1.0 nm minimum distance to edge) and neutralized with counter-ions. Following steepest descent energy minimization (Fmax < 1,000 kJ·mol^−1^·nm^−1^), the system was equilibrated through sequential 100 ps NVT (300 K) and 100 ps NPT (1 bar) simulations with positional restraints. The production MD was run with a 2 fs time step. Trajectory analysis included calculating RMSD, RMSF, Rg, SASA, and hydrogen bonds. Additionally, a free energy landscape (FEL) was constructed from the first two principal components (PC1, PC2) of the ligand's trajectory. All data were plotted using Origin 2024.

### 2.8 Statistical analysis

Data are expressed as mean ± SD from at least three independent biological replicates per group. Technical replicates within each biological replicate are averaged before statistical analysis. All statistical analyses were conducted using GraphPad Prism, version 9.5.0 (San Diego, CA, USA). For comparisons between two independent groups, an unpaired, two-tailed Student's *t*-test was applied. When comparing more than two groups under a single experimental factor, one-way analysis of variance (ANOVA) was performed, followed by Tukey's *post-hoc* test for multiple comparisons. Specifically, differences among the control group (HT22), the oxidative stress group (HT22 + H_2_O_2_), and the treatment group (HT22 + H_2_O_2_ + NGN) were evaluated using one-way ANOVA. The graphical abstract and mechanism diagram were drawn using Figdraw 2.0 tools (www.figdraw.com).

## 3 Results

### 3.1 Screening of candidate compounds and prediction of putative target proteins

**Figure 2:**
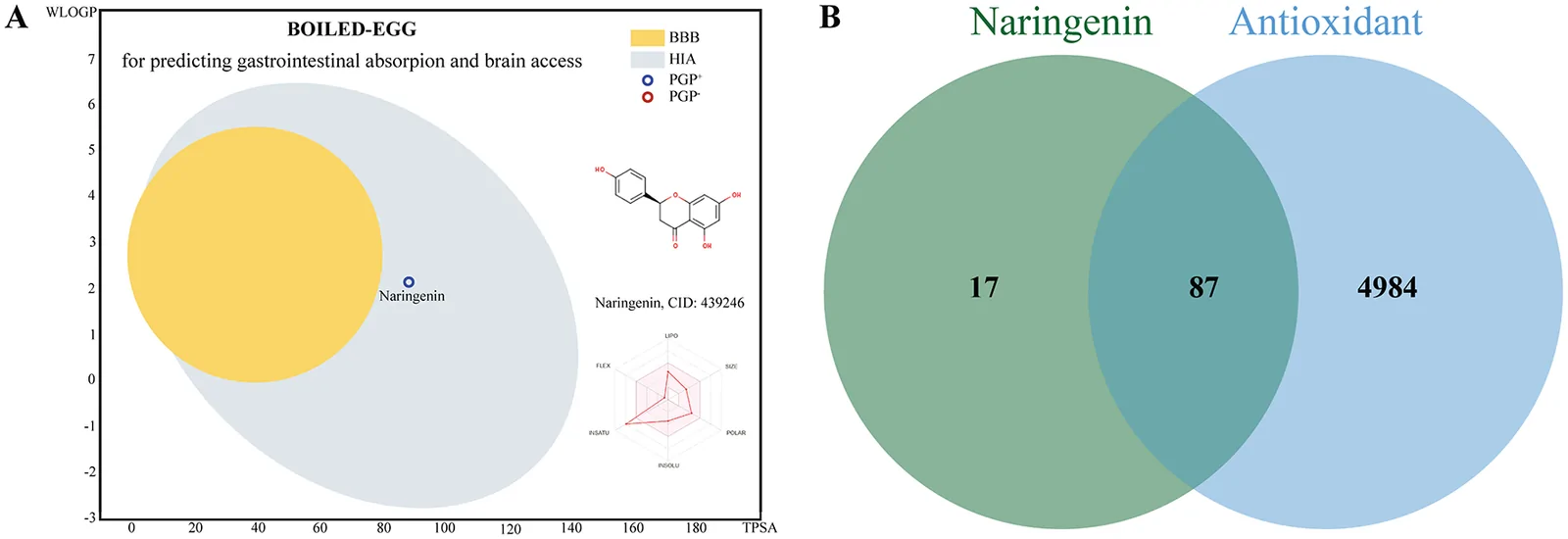
Pharmacokinetic properties and putative antioxidant targets of NGN. **(A)** Pharmacokinetic profile predicted by the SwissADME platform. **Left:** BOILED-Egg plot illustrating BBB permeability (yolk for high, white for low) and P-gp substrate status. **Right:** Bioavailability radar plot displaying the optimal physicochemical space (shaded area). **(B)** Venn diagram showing the intersection between the predicted NGN targets and the antioxidant-related gene set.

Naringenin (NGN) was predicted to possess favorable drug-like properties, characterized by high human intestinal absorption (HIA) but poor blood-brain barrier (BBB) permeability. The analysis also identified NGN as a substrate of P-glycoprotein [P-gp(+)], suggesting potential efflux-associated pharmacokinetic limitations (Figure 2A). To elucidate its molecular mechanism, 104 putative protein targets for NGN were identified through a consensus of the SwissTargetPrediction and Super-PRED databases. Intersecting these targets with 5,071 known antioxidant-related genes from GeneCards revealed that 87 of these proteins are implicated in antioxidant defense pathways, providing a strong basis for NGN's mechanism of action (Figure 2B, Supplementary Table 1).

### 3.2 Construction and analysis of an integrated network model

**Figure 3:**
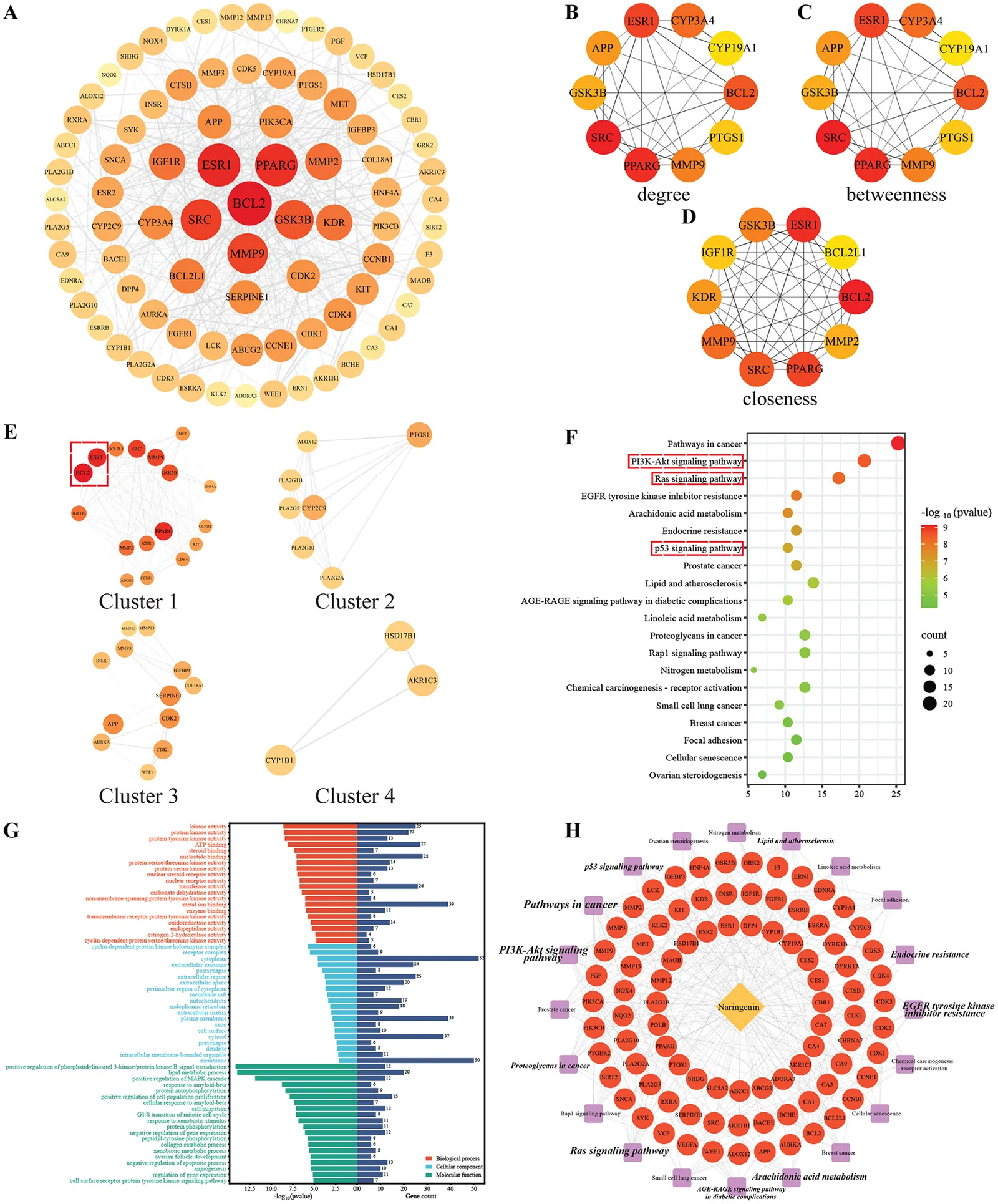
Integrated network and enrichment analysis of putative targets. **(A)** Protein-protein interaction (PPI) network. **(B–D)** Visualization of key network topological parameters: degree **(B)**, betweenness centrality **(C)**, and closeness centrality **(D)**. **(E)** Identification of highly interconnected modules via cluster analysis. **(F)** Bubble chart of the top 20 enriched KEGG pathways. **(G)** Bar charts displaying the top 10 enriched GO terms across biological processes, cellular components, and molecular functions. **(H)** The C-T-P interaction network.

To elucidate NGN's mechanism of action, a PPI network of the 87 identified antioxidant targets was constructed (Figure 3A). In network pharmacology analyses, core targets are primarily screened based on the topological centrality of PPI networks, with degree centrality being one of the most widely used metrics for identifying key proteins (49, 50). After ranking all candidate targets in descending order by degree centrality, we focused on the top-ranked key targets for downstream mechanistic studies, ultimately identifying key bottleneck proteins such as SRC, ESR1, PPARG, HO-1, GSK3B, MMP9, NRF2, and BCL-2 (Figures 3B–D). Among these, the top six targets by degree centrality were prioritized for experimental validation. Subsequent clustering revealed multiple functional modules (Figure 3E), with the primary cluster (Cluster 1) featuring highly connected bottleneck nodes like BCL-2 and ESR1. Furthermore, KEGG pathway enrichment analysis revealed that these 87 targets were significantly involved in core signaling cascades, particularly the PI3K-Akt, Ras, and p53 pathways (Figure 3F). Notably, the PI3K-Akt pathway exhibited the highest statistical significance. However, as a broad upstream signaling hub (51), the PI3K-Akt cascade coordinately regulates a variety of downstream effectors, including apoptosis, cell proliferation, and metabolism, rather than being exclusively associated with the oxidative stress response; this limits its specificity as a core protective mechanism underlying NGN's resistance to oxidative damage. A growing body of evidence suggests that the NRF2/HO-1 axis is a classic downstream antioxidant module activated by PI3K-Akt signaling in the neuronal environment (52, 53). Therefore, NRF2 and HO-1 were prioritized for experimental validation to obtain specific molecular evidence for NGN's neuroprotective mechanism. GO analysis characterized their functional roles, highlighting enrichment in biological processes such as kinase activity regulation, cellular components including neuronal axons, and molecular functions associated with signaling pathway modulation (Figure 3G). Finally, a compound-target-pathway (C-T-P) network was generated, systematically illustrating the multi-target and multi-pathway framework underlying NGN's potential antioxidant activity (Figure 3H).

### 3.3 Impedance spectrum parameters of HT22 cells

**Figure 4:**
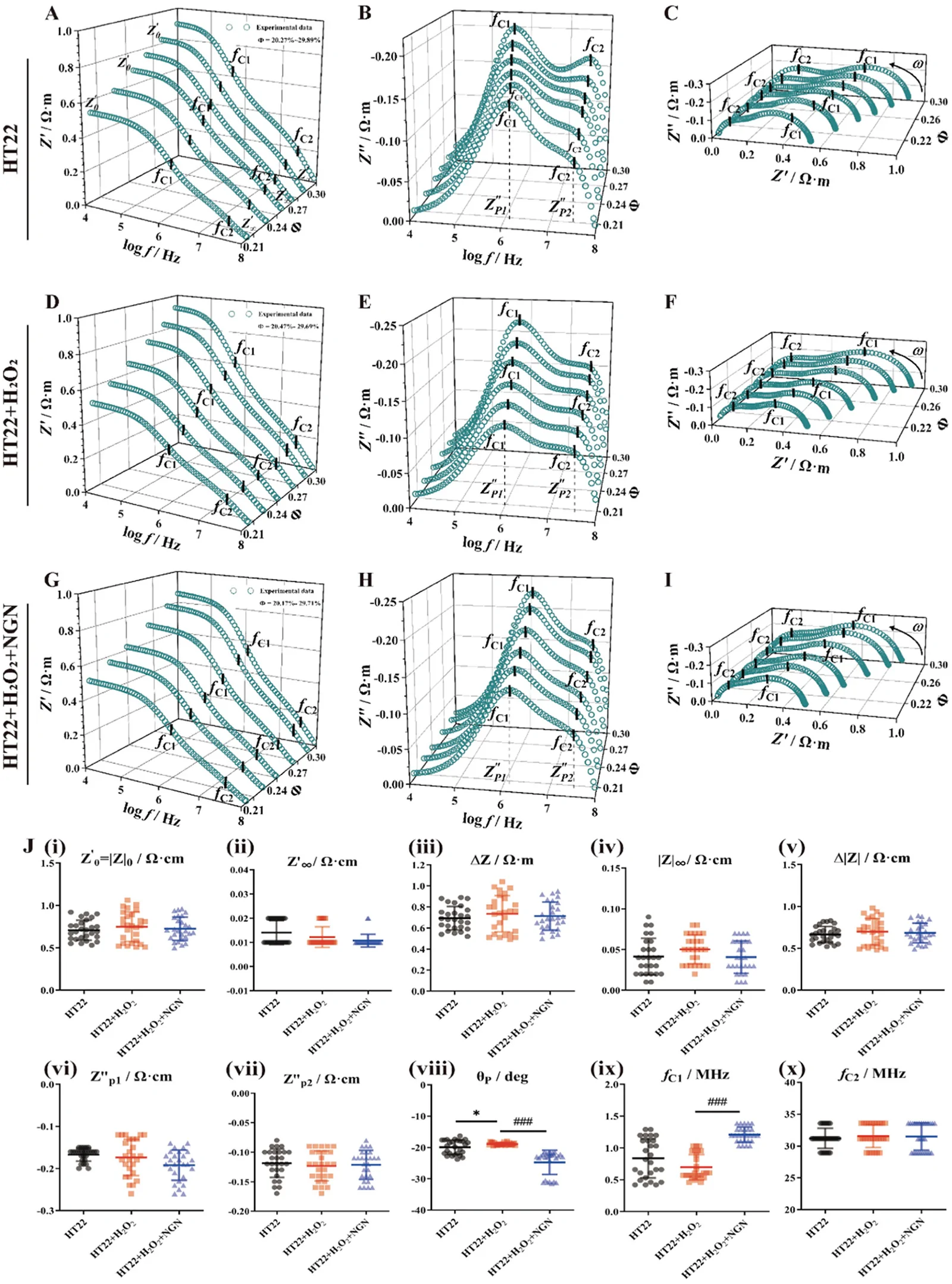
Impedance spectra and extracted parameters of HT22 cells across different treatment groups. **(A, D, G)** Real part and **(B, E, H)** imaginary part of the impedance spectra. **(C, F, I)** Nyquist plots. **(J)** Quantitative comparisons of the extracted impedance parameters (i-x). Green dots in **(A–I)** represent experimental data points. All impedance measurements were recorded over a frequency range of 10 kHz to 100 MHz at a controlled cell density (20%−35%). The evaluated groups include the untreated control (HT22), the oxidative damage model (HT22 + 200 μmol/L H_2_O_2_), and the NGN protection group (HT22 + 200 μmol/L H_2_O_2_ + 50 μmol/L NGN). For each concentration point within the tested range, data are presented as mean ± SD. *p < 0.05 vs. the HT22 control; ^###^*p* < 0.001 vs. the H_2_O_2_-treated group.

Cellular impedance spectroscopy was employed to assess the electrophysiological features of HT22 cells under oxidative stress and the neuroprotective effects of NGN. Representative impedance spectra across the 10 kHz to 100 MHz frequency range are summarized in Figure 4. The real part of the impedance (*Z*′−*f*) (Figures 4A, D, G) displayed typical capacitive behavior, maintaining stable high values at low frequencies (10–100 kHz) before progressively decreasing. As the frequency increased from 0.1 MHz to 10 MHz, *Z*′ decreased progressively, revealing two characteristic frequencies (*f*_*C*1_ and *f*_*C*2_) corresponding to polarization at the plasma membrane and nuclear membrane, respectively. At ultra-high frequencies (>10 MHz), *Z*′ approached the high-frequency limit ${Z^{\prime }}_{\infty }$, reflecting the electrical properties of nuclear and cytoplasmic contents. Compared to the control, H_2_O_2_ exposure elevated the Δ*Z*′ and Δ|*Z*| parameters, while NGN co-treatment effectively reversed these changes, restoring them toward baseline levels.

The imaginary part of the impedance (*Z*″) exhibited a characteristic double-peak profile separated by a single valley (Figures 4B, E, H). The first peak (0.1–1 MHz) corresponds to plasma membrane polarization, while the second peak (~10 MHz) reflects nuclear membrane polarization. Notably, H_2_O_2_ treatment specifically shifted the first characteristic frequency (*f*_*C*1_) to higher values, an effect that was significantly reversed by NGN. In contrast, the second characteristic frequency (*f*_*C*2_) remained largely unchanged across all groups, suggesting that oxidative damage predominantly targets the plasma membrane rather than nuclear structures. Nyquist plots (*Z*′ vs. *Z*″) further corroborated this, revealing depressed semicircular profiles with distinct group-specific variations in peak asymmetry, particularly evident in the relative amplitudes of the low-frequency component (Figures 4C, F, I).

Quantitative comparison of the extracted impedance parameters (Table 1, Figure 4J) further validated these dielectric alterations. Specifically, the phase angle (θ_*p*_) was significantly elevated in the H_2_O_2_ group (−18.89 ± 0.42 deg) compared to the control (−19.90 ± 2.28 deg, *p* < 0.05), but was normalized by NGN treatment [−24.74 ± 3.88 deg, Figure 4J(viii)]. Additionally, *f*_*C*1_ was dramatically decreased following H_2_O_2_ exposure and markedly restored by NGN [*p* < 0.001, Figure 4J(ix)]. Other key parameters, including low/high-frequency impedance magnitude (|*Z*|_0_, |*Z*|_∞_), real part increment (*Z*), and imaginary part peaks (${Z^{\prime \prime }}_{P1},{Z^{\prime \prime }}_{P2}$), showed consistent trends of H_2_O_2_-induced disruption and NGN-associated restoration [Figure 4J(i-vii)]. Collectively, these bioelectrical profiles demonstrate that H_2_O_2_ disrupts neuronal plasma membrane integrity, and NGN confers significant biophysical protection by restoring these parameters to near-physiological states.

**Table 1:** Impedance spectra parameters in HT22 cells under different treatment conditions.

Parameters	Symbol/unit	HT22	HT22 + H_2_O_2_	HT22 + H_2_O_2_ + NGN
Low frequency limit of real part of impedance	*Z*′_0_/Ω·m	0.71 ± 0.12	0.75 ± 0.17	0.72 ± 0.14
High frequency limit of real part of impedance	*Z*′_∞_/Ω·m	0.01 ± 0.00	0.01 ± 0.00	0.01 ± 0.00
Real part increment of electrical impedance	Δ*Z* /Ω·m	0.69 ± 0.11	0.74 ± 0.17	0.71 ± 0.13
Impedance amplitude at high frequency	|*Z*|_∞_/Ω·m	0.04 ± 0.02	0.05 ± 0.02	0.04 ± 0.02
Impedance amplitude increment	Δ|*Z*|/Ω·m	0.67 ± 0.09	0.70 ± 0.16	0.68 ± 0.12
Peak of imaginary part of impedance 1	*Z*″_*p*1_Ω·m	−0.17 ± 0.02	−0.17 ± 0.04	−0.19 ± 0.04
Peak of imaginary part of impedance 2	*Z*″_*p*2_/Ω·m	−0.12 ± 0.02	−0.12 ± 0.02	−0.12 ± 0.02
Peak of phase angle	θ_P_/deg	−19.90 ± 2.28	−18.89 ± 0.42^*^	−24.74 ± 3.88^###^
The 1st characteristic frequency	*f*_*C*1_/MHz	0.84 ± 0.31	0.70 ± 0.20	1.21 ± 0.12^###^
The 2nd characteristic frequency	*f*_*C*2_/MHz	31.23 ± 1.57	31.59 ± 1.79	31.52 ± 2.16

Definitions: ${Z^{\prime }}_{0}=|Z{|}_{0};Z={Z^{\prime }}_{0}-{Z^{\prime }}_{\infty };\left|Z\right|=|Z{|}_{0}-|Z{|}_{\infty }$. For each concentration point within the tested range, data are presented as mean ± SD. ^*^p < 0.05 vs. the HT22 control; ^#^*p* < 0.05 vs. the H_2_O_2_-treated group.

### 3.4 Model fitting and parameter extraction

**Figure 5:**
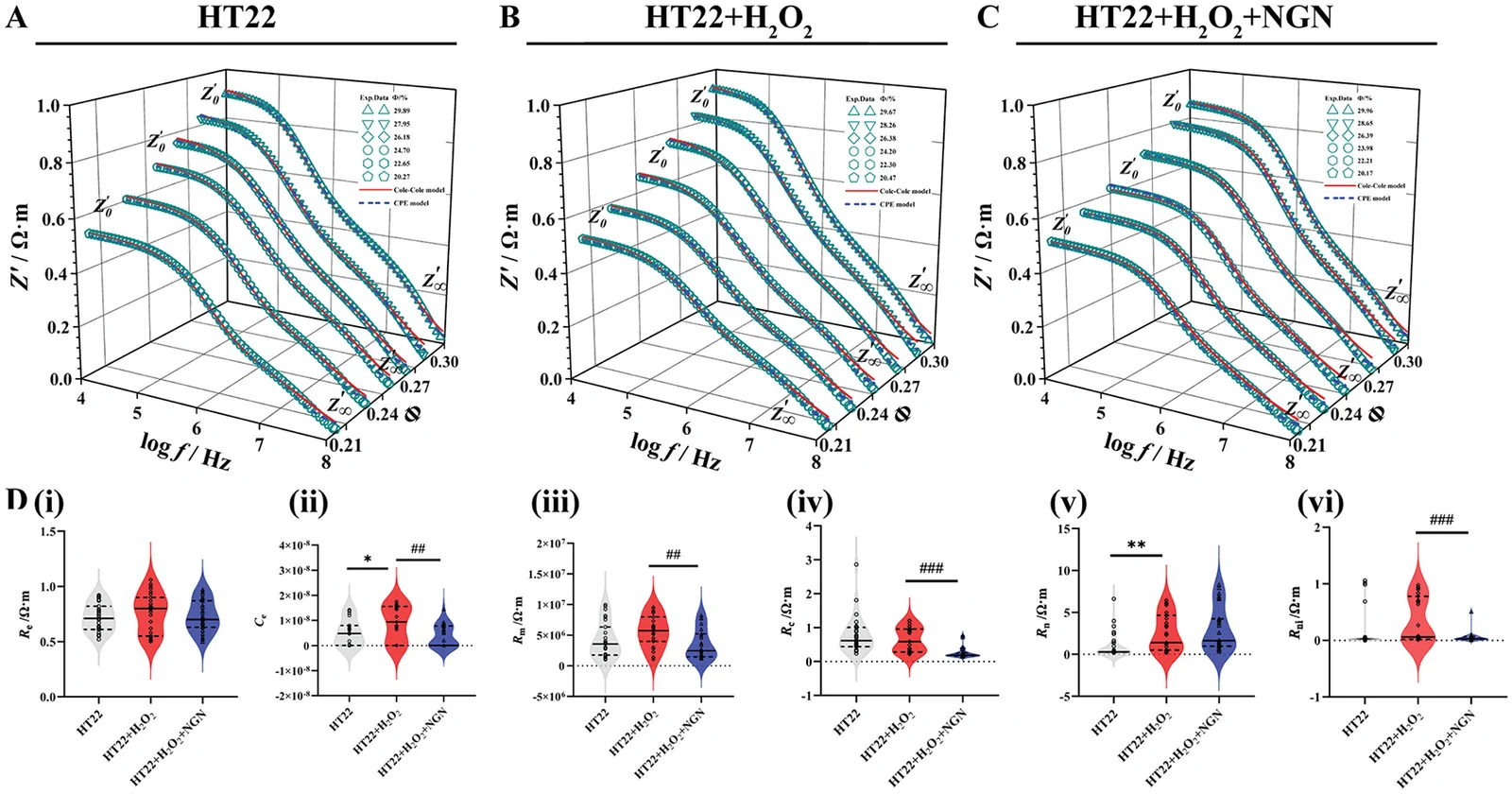
Dual-model fitting of impedance spectra and extracted dielectric parameters. **(A, B, C)** Real part of the impedance spectra. In **A–C**, green hollow circles represent experimental data, while red solid lines and blue dashed lines indicate fits derived from the Cole–Cole mathematical model and the CPE-based equivalent circuit model, respectively. **(D)** Specific cellular electrical parameters derived from the CPE-based equivalent circuit model. (i) Extracellular capacitance (*R*_*e*_). (ii) Extracellular capacitance (*C*_*e*_). (iii) Cell membrane resistance (*R*_*m*_). (iv) Cytoplasmic resistance (*R*_*c*_). (v) Nuclear resistance (*R*_*n*_). (vi) Intranuclear resistance (R_ni_). For each concentration point within the tested range, data are presented as mean ± SD. *p < 0.05, **p < 0.01 vs. the HT22 control group; ^##^*p* < 0.01, ^###^*p* < 0.001 vs. the H_2_O_2_-treated group.

To quantitatively interpret the electrophysiological alterations, the experimental impedance spectra were fitted to both a bi-disperse Cole–Cole model and an eight-element constant phase element (CPE)-based equivalent circuit model. Both models demonstrated excellent agreement with the measured data across all experimental groups (Figures 5A–C, Supplementary Figure 1). Cole–Cole parameter extraction (Supplementary Table 2) revealed that H_2_O_2_ exposure significantly decreased both characteristic frequencies compared to the control [*f*_C1_ = (5.60 ± 1.16) × 105 Hz vs. 6.82 ± 2.41 × 105 Hz; *f*_C2_ = 3.01 ± 0.44 × 107 Hz vs. 3.40 ± 0.51 × 107 Hz]. Notably, NGN co-treatment reversed the H_2_O_2_-induced decline in *f*_C1_ (recovering to 9.68 ± 0.47 × 105 Hz) but paradoxically further reduced *f*_C2_ (2.51 ± 0.70 × 107 Hz).

Concurrently, analysis derived from the CPE equivalent circuit model (Figure 5D, Table 2) demonstrated that H_2_O_2_ significantly elevated the extracellular capacitance (*C*_*e*_) and nuclear resistance (*R*_*n*_), reflecting disrupted extracellular ionic homeostasis and impaired nuclear envelope integrity. NGN treatment effectively attenuated these specific H_2_O_2_-induced elevations while simultaneously normalizing other critical dielectric components, including the nuclear dispersion coefficient (α_*n*_), membrane and nuclear pseudo-capacitances (*CPE*_*Tm*_, *CPE*_*Tn*_), and various resistive components (*R*_*m*_, *R*_*ni*_, *R*_*c*_), thereby restoring the lipid bilayer structure and intracellular bioelectrical environment. Collectively, these dual-model extractions quantitatively confirm that NGN ameliorates oxidative damage by actively restoring neuronal membrane integrity and intracellular bioelectrical properties.

**Table 2:** Quantitative cellular electrical [[Inline Image]]parameters derived from the CPE-based equivalent circuit modeling of HT22 cells.

Circuit model parameters	Symbol/unit	HT22	HT22 + H_2_O_2_	HT22 + H_2_O_2_ + NGN
Extracellular capacitance	*C*_*e*_	(0.51 ± 0.53) × 10^−8^	(0.88 ± 0.71) × 10^−8*^	(0.36 ± 0.43) × 1 0^−8*##*^
Extracellular resistance	*R*_*e*_/Ω·m	0.72 ± 0.12	0.76 ± 0.18	0.73 ± 0.14
Pseudo-capacitance of cell membrane	*CPE*_*Tm*_/nF$\cdot {\text{s}}^{\alpha_{m}-1}$	(1.90 ± 0.20) × 10^−4^	(1.97 ± 0.25) × 10^−4^	(0.75 ± 0.03) × 10^−4#^
Dispersion coefficient of cell membrane	α_*m*_	0.74 ± 0.08	0.76 ± 0.09	0.75 ± 0.03
Cell membrane resistance	*R*_*m*_/Ω·m	(4.15 ± 2.87) × 10^6^	(5.59 ± 2.65) × 10^6^	(3.46 ± 2.45) × 10^6##^
Pseudo-capacitance of cell nucleus	*CPE*_*Tn*_/ nF$\cdot {\text{s}}^{\alpha_{m}-1}$	(9.19 ± 26.72) × 10^−7^	(9.54 ± 19.05) × 10^−7^	(0.01 ± 0.01) × 10^−7#^
Dispersion coefficient of cell nucleus	α_*n*_	1.05 ± 0.19	1.02 ± 0.23	1.23 ± 0.12^###^
Nucleus resistance	*R*_*n*_/Ω·m	1.05 ± 1.54	2.53 ± 2.19^**^	3.00 ± 2.63
Intranuclear resistance	*R*_*ni*_/Ω·m	0.16 ± 0.33	0.36 ± 0.39	0.06 ± 0.09^###^
Cytoplasmic resistance	*R*_*c*_/Ω·m	0.78 ± 0.56	0.64 ± 0.35	0.26 ± 0.16^###^
Chi-Sqr	χ^2^	0.02 ± 0.01	0.01 ± 0.01^*^	0.02 ± 0.01
Sum-Sqr	∑χ^2^	3.85 ± 2.32	2.74 ± 1.29^*^	3.71 ± 2.34

For each concentration point within the tested range, data are presented as mean ± SD. ^*^p < 0.05, ^**^p < 0.01 vs. the HT22 control; ^#^*p* < 0.05, ^##^*p* < 0.01, ^###^*p* < 0.001 vs. the H_2_O_2_-treated group.

### 3.5 Biochemical verification of the antioxidant effects of NGN on H_2_O_2_-induced HT22 cells

**Figure 6:**
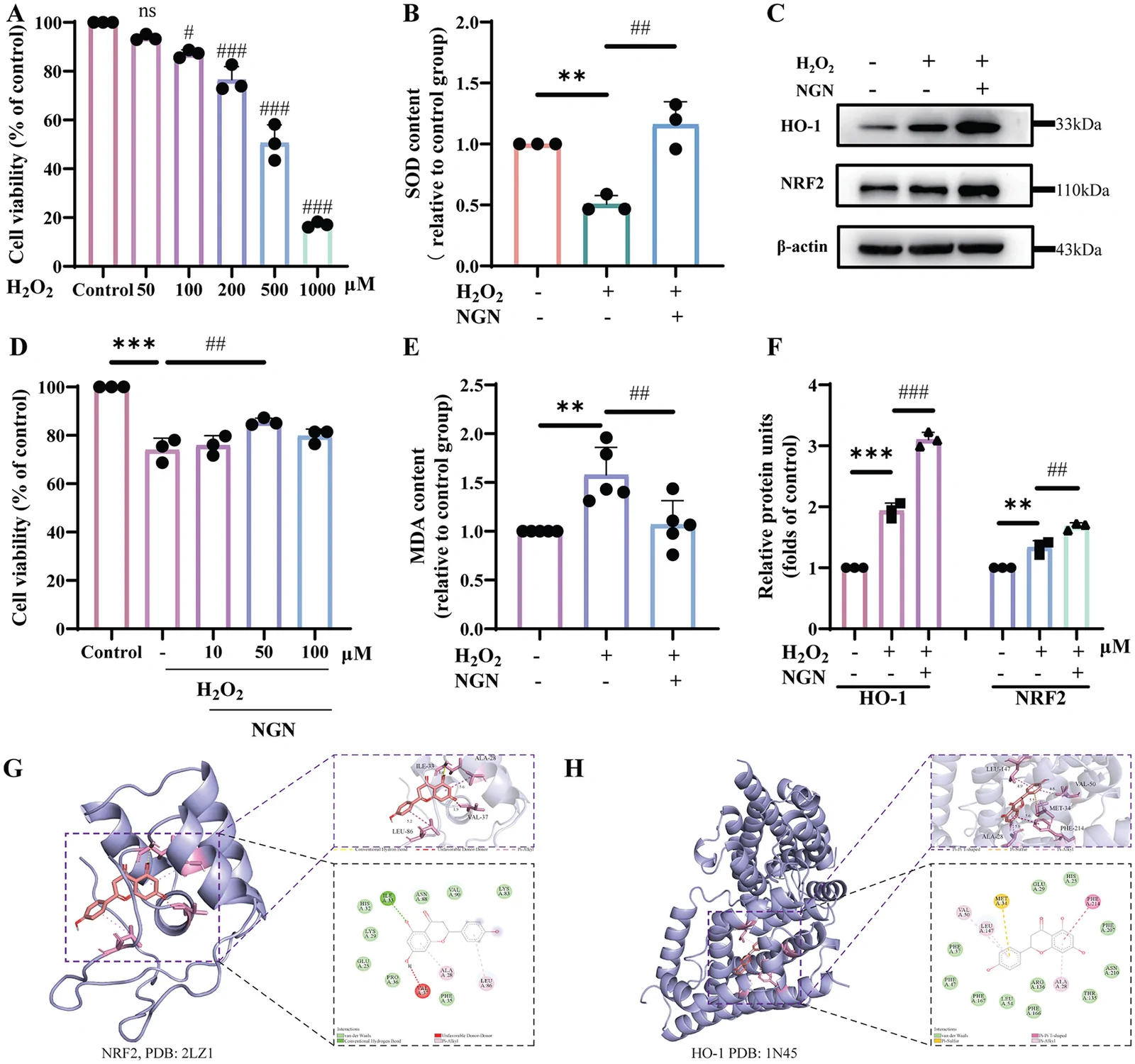
Biochemical validation of the antioxidant and neuroprotective effects of NGN in HT22 cells. **(A)** Cell viability following H_2_O_2_ exposure. **(B)** Protective effect of NGN on cell viability under H_2_O_2_-induced oxidative stress. **(C, D)** Effects of NGN on **(C)** SOD activity and **(D)** MDA accumulation. **(E)** Western blot analysis and **(F)** relative quantification of NRF2 and HO-1 protein expression levels. **(G, H)** Molecular docking simulations illustrating the binding interactions between NGN and both NRF2 and HO-1. Data are presented as mean ± SD from at least three independent biological replicates per condition. **p < 0.01, ***p < 0.001 vs. the HT22 control group; ^#^*p* < 0.05, ^##^*p* < 0.01, ^###^*p* < 0.001 vs. the H_2_O_2_-treated group. ns, not significant (*p*> 0.05).

To biochemically validate the neuroprotective potential suggested by impedance assays, we evaluated cellular responses to oxidative stress. Treatment with NGN (50 μmol/L) significantly reversed the severe reduction in cell viability induced by 24 h exposure to 200 μmol/L H_2_O_2_ (Figures 6A, B). Furthermore, NGN effectively restored the activity of the antioxidant enzyme superoxide dismutase (SOD, Figure 6C) and suppressed the accumulation of malondialdehyde (MDA, Figure 6D), confirming its potent capacity to mitigate lipid peroxidation. Guided by our network pharmacology findings, we investigated the NRF2/HO-1 signaling axis as a pivotal hub for this antioxidant defense. Western blot analysis revealed that while H_2_O_2_ exposure triggered a compensatory increase in NRF2 and HO-1 protein levels, NGN co-treatment robustly amplified the expression of both proteins beyond the H_2_O_2_-induced baseline (Figures 6E, F). To explore the structural basis underlying these effects, molecular docking simulations were conducted. The results demonstrated that NGN forms highly stable interactions with NRF2 (binding energy: −6.335 kcal/mol, Figure 6G) and HO-1 (−7.976 kcal/mol, Figure 6H), as well as with other key network targets including SRC (−8.035 kcal/mol), ESR1 (−8.023 kcal/mol), PPARG (−7.987 kcal/mol), GSK3β (−7.976 kcal/mol), MMP9 (−6.565 kcal/mol), and BCL-2 (−5.728 kcal/mol) (Supplementary Figure 2). Collectively, these *in vitro* and in silico data provide compelling evidence that NGN exerts its neuroprotective and antioxidant effects primarily through the direct engagement and robust activation of the NRF2/HO-1 pathway.

### 3.6 Multi-parameter validation of NGN-target protein complex stability via MD simulations

**Figure 7:**
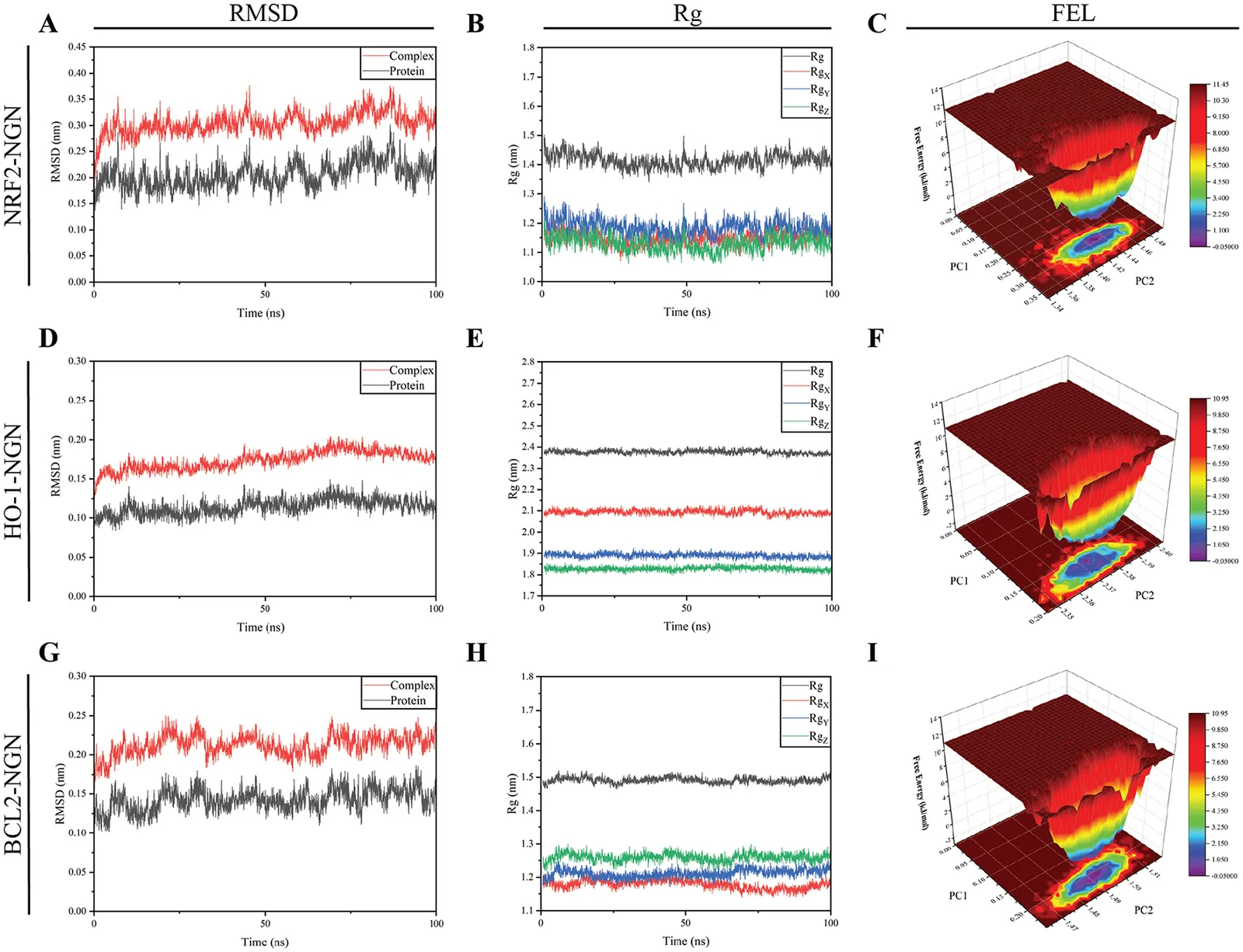
Multiparametric analysis of NRF2-NGN **(A–C)**, HO-1-NGN **(D–F)**, and BCL2-NGN **(G–I)** stability via molecular dynamics modeling. **(A, D, G)** Time - resolved root mean square deviation (RMSD) plots; **(B, E, H)** Radius of gyration (Rg) variations; **(C, F, I)** Free energy landscapes (FELs).

To validate the dynamic binding stability of NGN with its core targets under physiological conditions, 100 ns molecular dynamics simulations were performed for the eight protein-ligand complexes (Figure 7, Supplementary Figure 3 and Supplementary Figure 4). Trajectory analyses revealed that all systems rapidly reached and maintained structural equilibrium, exhibiting low average root mean square deviations (RMSD < 0.6 nm) and minimal radius of gyration (Rg) fluctuations (SD ≤ 0.024 nm) (Supplementary Table 3). Notably, the BCL2-NGN and NRF2-NGN complexes displayed the most compact conformations. Root mean square fluctuation (RMSF) and solvent-accessible surface area (SASA) profiles further confirmed this overall stability, showing rigid structural cores and stable binding pockets, with flexibility confined appropriately to unbound loop regions. Intermolecular hydrogen bond tracking highlighted robust polar interactions driving these affinities, particularly within the BCL2 (Supplementary Figure 3I) and MMP9 [Supplementary Figure 4E(v)] complexes, which consistently maintained the highest number of stable hydrogen bonds. Finally, free energy landscape (FEL) analyses demonstrated that most complexes settled into a single, deep, low-energy basin, indicative of a dominant and tightly locked binding conformation. Interestingly, the ESR1 complex exhibited a dual-well FEL configuration [Supplementary Figure 4B(vi)], suggesting the existence of two stable states and potential conformational switching. Collectively, these multiparametric thermodynamic data firmly corroborate the molecular docking predictions. Importantly, the exceptional dynamic stability observed for NGN bound to core targets—particularly NRF2, HO-1, and BCL2—provides robust *in silico* evidence supporting our *in vitro* biochemical findings. This confirms, at the atomic level, that NGN exerts its potent neuroprotective and antioxidant effects primarily by stably engaging and modulating the NRF2/HO-1 signaling pathway.

## 4 Discussion

This study elucidates the neuroprotective mechanisms of NGN—a principal bioactive flavonoid derived from *Citrus reticulata* peel (CRP)—against H_2_O_2_-induced oxidative injury in HT22. By integrating electrical impedance spectroscopy (EIS) with biophysical modeling, network pharmacology, and multi-scale computational simulations, we provide a multidimensional perspective on NGN's protective actions. Our findings reveal a coherent pharmacological mechanism where NGN actively engages the NRF2/HO-1 antioxidant axis, subsequently preserving neuronal membrane electro-biophysical integrity.

A notable innovation of this work lies in the utilization of EIS coupled with Cole–Cole and an eight-element CPE-based circuit model to achieve a quantitative, non-invasive, and real-time assessment of neuronal membrane integrity at subcellular resolution (39, 54). Our impedance spectra revealed two distinct characteristic frequencies: a low-frequency peak (*f*_*C*1_) associated with plasma membrane polarization and a high-frequency peak (*f*_*C*2_) reflecting nuclear membrane dynamics. Oxidative stress led to altered membrane capacitance (*C*_*m*_) and increased membrane resistance (*R*_*m*_). NGN treatment significantly mitigated these H_2_O_2_-induced dielectric disruptions, specifically normalizing *f*_*C*1_ and restoring phase angle peaks. This demonstrates that NGN's primary physical protective site is the plasma membrane. In our model, *f*_*C*2_ is associated with the dielectric relaxation of the nuclear envelope. Under H_2_O_2_-induced oxidative stress, nuclear membrane damage—including lipid peroxidation and perinuclear space expansion—typically elevates the local dielectric constant, resulting in a higher characteristic frequency. We propose that NGN's robust antioxidant activity alleviates this damage, restoring a more compact and electrochemically stable nuclear membrane configuration. This biophysical normalization would consequently shift *f*_*C*2_ to a lower value, this reduction is a positive indicator of membrane recovery, rather than a contradictory functional impairment. We acknowledge that high-frequency fitting inherently carries greater uncertainty due to electrode polarization and reduced signal-to-noise ratio in the kilohertz–megahertz range. In future research, conducting rigorous structural validation—such as TEM assessment of nuclear morphology—would be valuable. These EIS-derived metrics provide an ultrasensitive, label-free biophysical readout of membrane repair, surpassing conventional biochemical endpoint assays, while establishing EIS as a robust tool for probing neuronal membrane health under stress.

In parallel, network pharmacology screening initially identified NGN as a high-priority neuroprotective candidate derived from CRP, demonstrating the power of this approach in deciphering the “multi-target, multi-pathway” complexity of traditional medicines. Although NGN exhibits moderate blood–brain barrier permeability (55), suggesting its potential for central nervous system activity (56, 57), its robust antioxidant efficacy—as evidenced by elevated SOD and HO-1 levels and reduced MDA content in our *in vitro* assays—highlights its therapeutic promise despite this pharmacokinetic limitation. Advanced delivery strategies, including nanoparticle encapsulation, have demonstrated substantial improvement in the brain delivery efficiency of flavonoids sharing similar physicochemical characteristics (58). Therefore, future investigations exploring NGN derivatives or targeted delivery systems may offer a viable approach to simultaneously preserve its neuroprotective potency and improve its cerebral bioavailability, thereby maximizing its therapeutic benefits in neurodegenerative diseases.

Notably, biochemical assays, molecular docking, and molecular dynamics simulations have collectively converged on the NRF2/HO-1 pathway as a key effector of NGN's action. However, a direct causal relationship between the two remains to be established. Systematic validation of this regulatory network through future gene knockdown or rescue experiments will help to further elucidate the causal mechanisms underlying the neuroprotective effects of NGN. Furthermore, other high-confidence targets predicted by network pharmacology analysis—including the estrogen receptor ESR1 (59) and the protein tyrosine kinase SRC (60)—have not yet been experimentally validated in this study. Future systematic experimental validation of these candidate targets will not only corroborate the reliability of the predictions from the network pharmacology screening in this study, but will also enable the construction of a more comprehensive framework for the multi-target neuroprotective mechanisms of NGN, thereby effectively addressing the inherent pathological complexity of neurodegenerative diseases.

To provide a definitive structural basis for these observations, 100-ns MD simulations were employed to substantiate NGN-target interactions under near-physiological kinetic conditions. Key thermodynamic parameters, including RMSD and Rg, confirmed that NGN forms exceptionally stable and compact complexes with core targets like NRF2, HO-1, and BCL-2. Furthermore, free energy landscape (FEL) analyses revealed that most NGN-target complexes settle into a single, deep low-energy basin, indicating a tightly locked, dominant binding conformation driven by robust intermolecular hydrogen bonding (particularly evident in the BCL-2 and MMP9 complexes). Interestingly, the dual-well FEL observed for the ESR1 complex suggests potential functional conformational switching. These kinetic profiles are highly consistent with recent computational studies, which have similarly demonstrated NGN's capacity to maintain stable, low-fluctuation interactions (characterized by low RMSD and Rg values) with isolated neurological or metabolic targets, such as MAO-B (61) and APOB (62). However, while previous *in silico* investigations have predominantly focused on single-receptor paradigms, our study advances the field by providing a comprehensive, multi-target thermodynamic landscape. By validating sustained hydrogen-bonding and compact folding across an entire antioxidant network, these robust *in silico* data strongly corroborate our *in vitro* findings, confirming that NGN's multi-targeted neuroprotection is underpinned by highly stable physicochemical binding affinities.

Collectively, this work elucidates a dual neuroprotective mechanism for NGN: biochemical enhancement of antioxidant capacity via direct NRF2/HO-1 pathway activation, and biophysical preservation of neuronal membrane integrity. These findings validate the traditional therapeutic utility of CRP and highlight NGN as a promising lead compound for managing broad oxidative stress-associated neurodegenerative disorders, such as Alzheimer's and Parkinson's diseases. Although the H_2_O_2_-induced HT22 model is a robust and well-established *in vitro* experimental system for studying widespread oxidative damage and redox imbalance, we acknowledge that this model cannot fully replicate the pathological environment of human neurodegenerative diseases. Therefore, future studies should evaluate the efficacy of NGN using more physiologically relevant disease-driven models, such as Aβ oligomer-induced or α-synuclein-induced neurotoxicity, to comprehensively validate the therapeutic significance of the NRF2/HO-1-associated neuroprotective effects identified in this study. Such investigations will not only further confirm the neuroprotective potential of NGN, but also allow for the assessment of its broader applicability in neurodegenerative diseases, including Alzheimer's disease and Parkinson's disease, and ultimately provide a more solid foundation for its clinical translational value. Furthermore, NGN possesses notable potential as a lead compound for developing multi-target therapeutic strategies against neurodegenerative disorders. Overall, this multidisciplinary strategy, featuring EIS for quantitative membrane evaluation, establishes a robust framework for pharmacomechanistic research and underscores the value of bridging atomic-level simulations, biochemical pathways, and macroscopic biophysical properties in neuroprotection studies.

## 5 Conclusions

In summary, our study provides novel mechanistic insights into the neuroprotective effects of naringenin (NGN) against oxidative stress. We have identified a dual mechanism of action: the reinforcement of cellular antioxidant pathways and the restoration of neuronal membrane biophysical integrity. Our primary finding, derived from a novel Electrical Impedance Spectroscopy (EIS) circuit model, is that NGN significantly reverses H_2_O_2_-induced membrane damage. This is evidenced by the recovery of key electro-biophysical parameters (Rm, Cm, and *f*_*C*1_), demonstrating a direct stabilizing effect on the neuronal membrane. This biophysical restoration is complemented by NGN's biochemical activity, where it potently activates the NRF2/HO-1 axis, boosts SOD levels, and reduces lipid peroxidation. Molecular simulations further support these findings, revealing stable, high-affinity binding of NGN to NRF2, HO-1, and BCL-2, which underpins its protective functions. A key contribution of this research is the successful application of EIS as a quantitative, label-free method to monitor neuronal membrane health at subcellular resolution. Collectively, by bridging traditional pharmacology with contemporary biophysical and *in silico* approaches, this research not only validates NGN as a promising therapeutic candidate for oxidative neurodegeneration but also establishes EIS as a highly sensitive, label-free tool for evaluating neuroprotective efficacy through the lens of electro-biophysical homeostasis.

## Data Availability

The original contributions presented in the study are included in the article/Supplementary material, further inquiries can be directed to the corresponding author.
