# Supplementary material for: Naringenin restores neuronal membrane electro-biophysical homeostasis: insights from EIS circuit modeling and molecular dynamics
Source: Front Med (Lausanne). 2026 Aug 31;13:1861895. doi: 10.3389/fmed.2026.1861895 (PMC13572258; doi:10.3389/fmed.2026.1861895)
Supplement: Supplementary file 1 [file Supplementary_file_1.pdf]

Supplementary Material

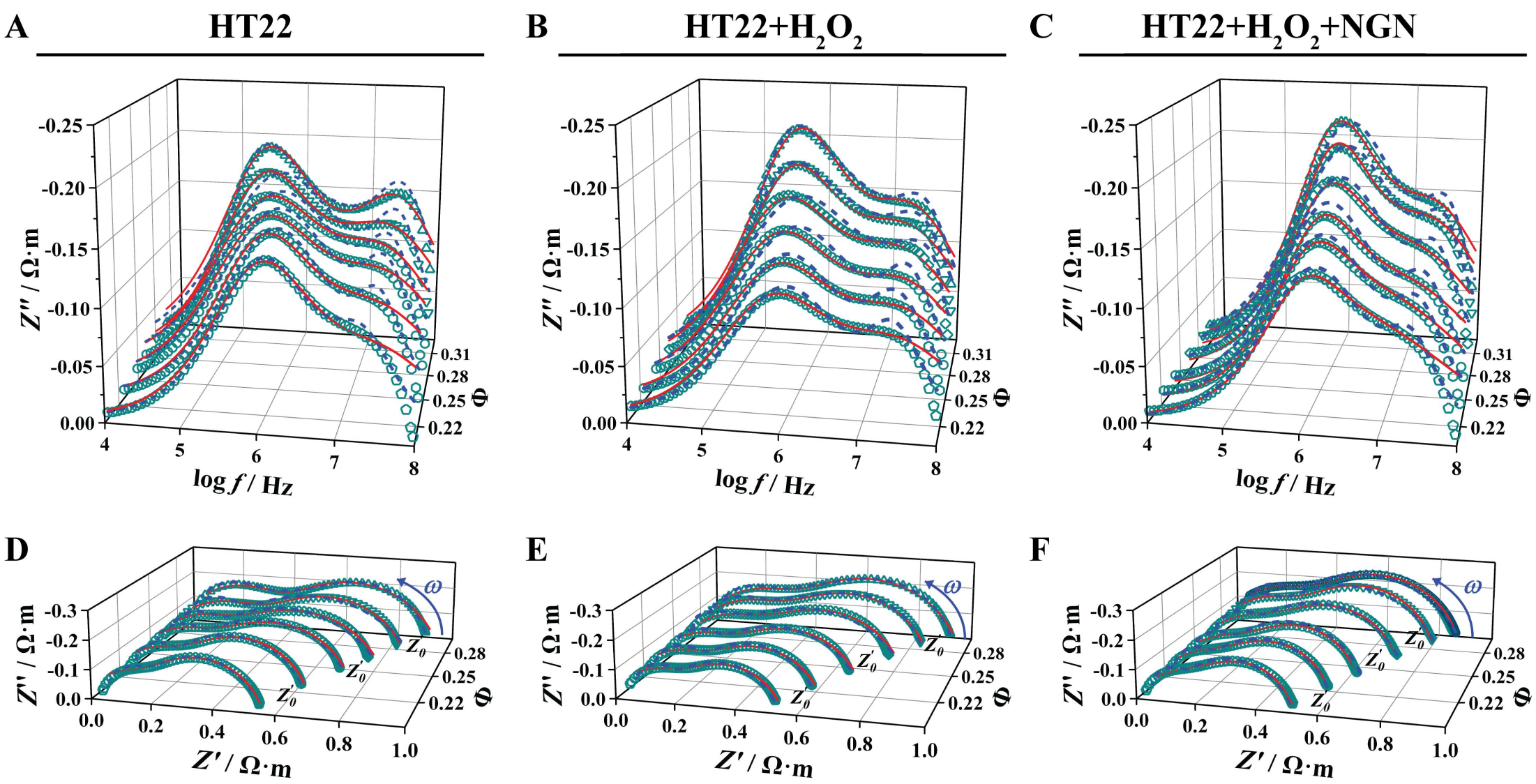

Supplementary Figure 1. Dual-model fitting of impedance spectra and extracted impedance parameters. (A, B, C) Imaginary impedance spectra. (D, E, F) Nyquist plots. Green hollow circles in A–F are experimental data; red solid lines and blue dashed lines show fits from the Cole-Cole model and the CPE-based equivalent circuit model, respectively.

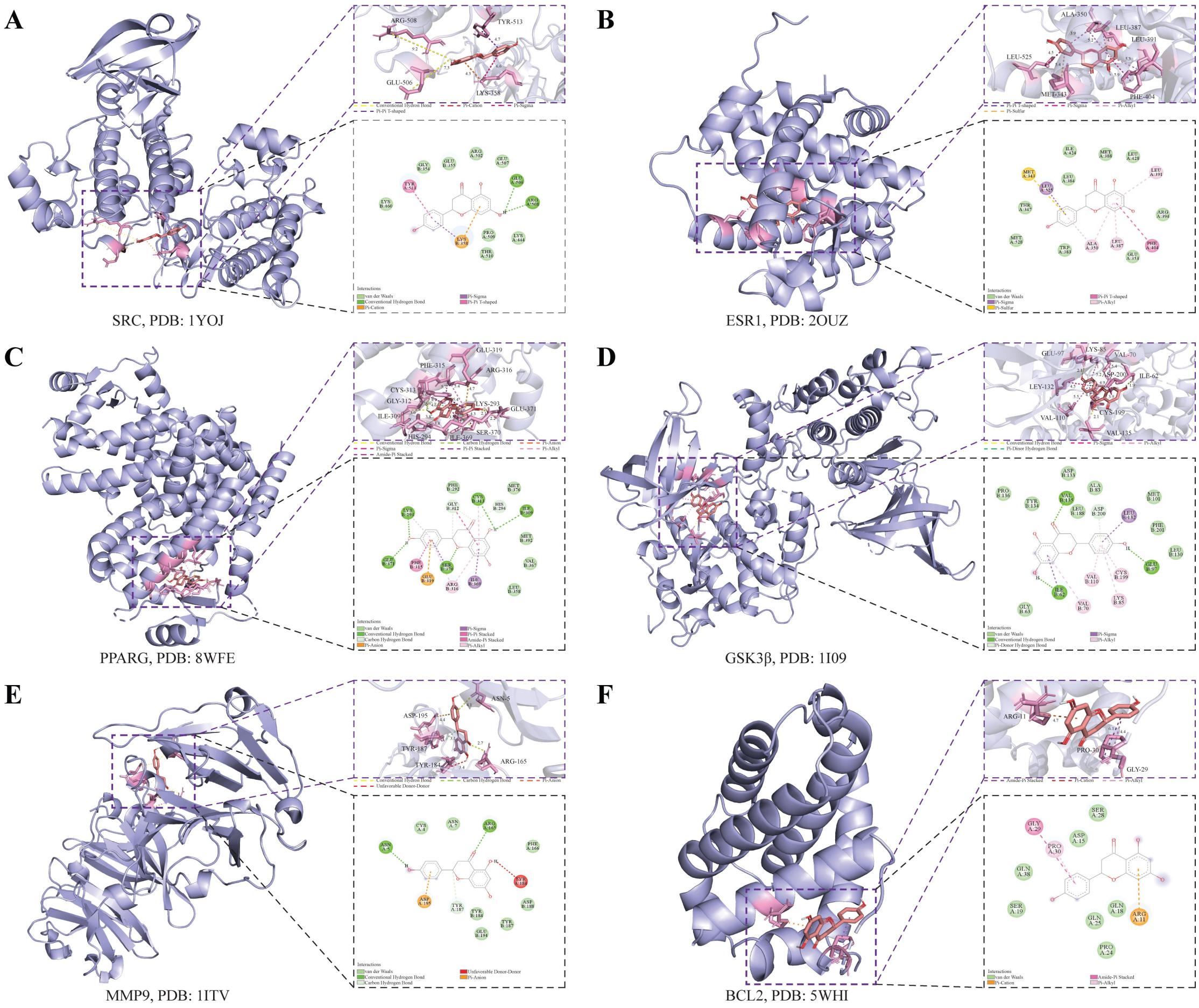

Supplementary Figure 2. Molecular docking simulations illustrating the binding interactions between NGN and SRC (A), ESR1 (B), PPARG (C), GSK3β (D), MMP9 (E) and BCL2 (F).

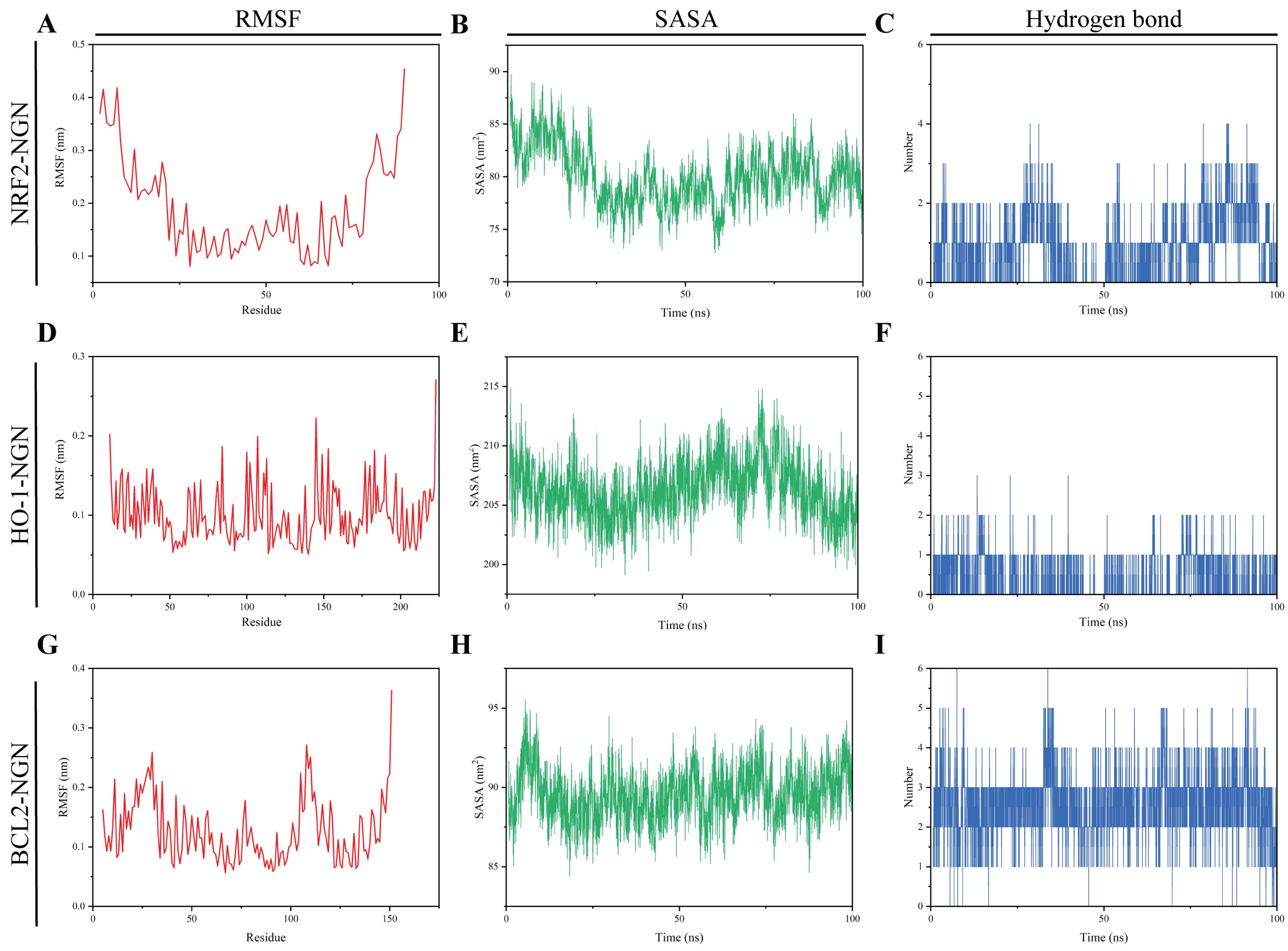

Supplementary Figure 3. Multiparametric analysis of NRF2-NGN (A-C), HO-1-NGN (D-F) and BCL2-NGN (G-I) stability via molecular dynamics modeling. (A, D, G) Root mean square fluctuations (RMSF); (B, E, H) Solvent - accessible surface area (SASA) profiles; (C, F, I) Intermolecular hydrogen bond counts over time.

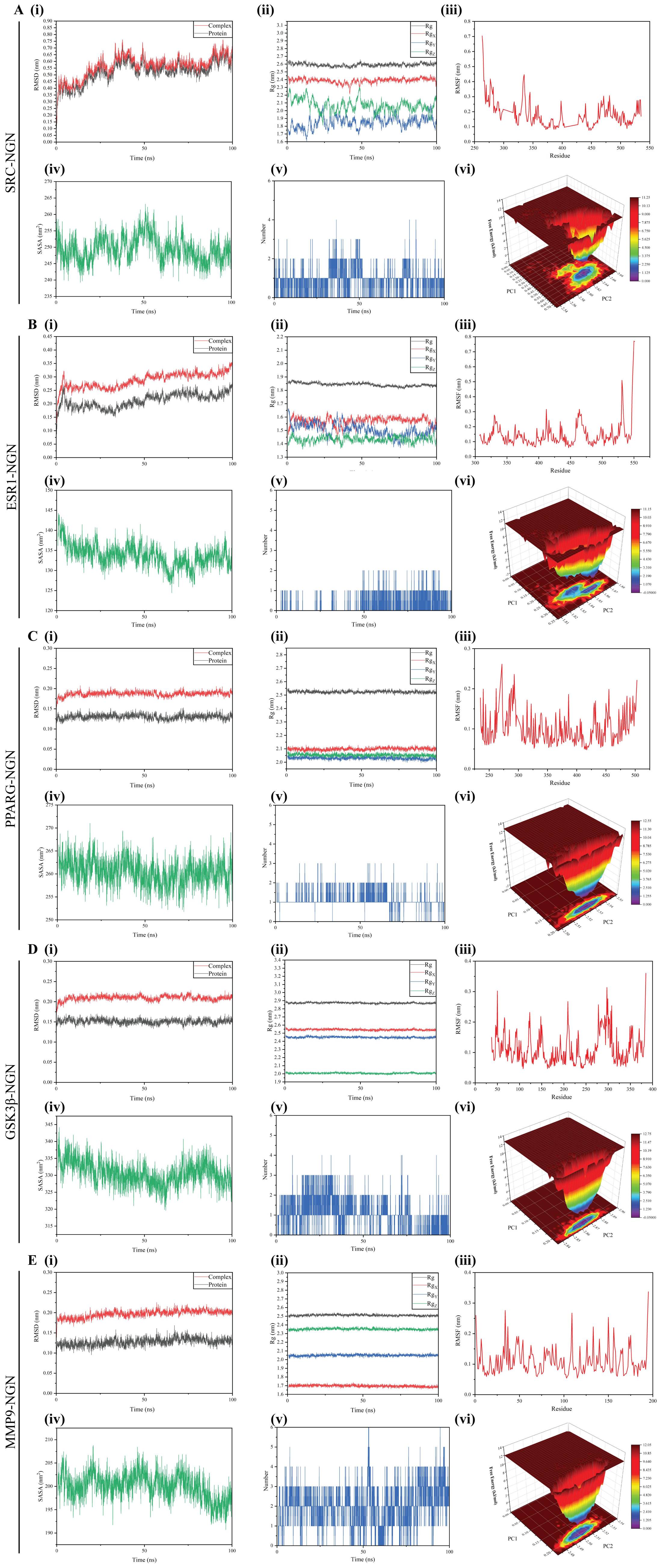

Supplementary Figure 4. Multiparametric analysis of SRC-NGN (A), ESR1-NGN (B), PPARG-NGN (C), GSK3β-NGN (D) and MMP9-NGN (E) stability via molecular dynamics modeling. (i) Time - resolved root mean square deviation (RMSD) plots; (ii) Radius of gyration (Rg) variations; (iii) Root mean square fluctuations (RMSF); (iv) Solvent - accessible surface area (SASA) profiles; (v) Intermolecular hydrogen bond counts over time; (vi) Free energy landscapes (FELs).

Supplementary Table 1. Potential Targets for NGN Antioxidant Defence.

Uniprot ID	Gene Symbol	Description	Category	Gifts	GC Id	Relevance score
P27487	DPP4	Dipeptidyl Peptidase 4	Protein Coding	63	GC02M161992	2.05456018447876
P11511	CYP19A1	Cytochrome P450 Family 19 Subfamily A Member 1	Protein Coding	60	GC15M051208	2.09918069839478
P43166	CA7	Carbonic Anhydrase 7	Protein Coding	50	GC16P066844	0.386310279369354
P33527	ABCC1	ATP Binding Cassette Subfamily C Member 1 (ABCC1 Blood Group)	Protein Coding	61	GC16P015949	2.62654376029968
P14061	HSD17B1	Hydroxysteroid 17-Beta Dehydrogenase 1	Protein Coding	54	GC17P175630	0.546325206756592
P04278	SHBG	Sex Hormone Binding Globulin	Protein Coding	55	GC17P007613	2.42663621902466
P22748	CA4	Carbonic Anhydrase 4	Protein Coding	58	GC17P176055	0.417015761137009
Q16678	CYP1B1	Cytochrome P450 Family 1 Subfamily B Member 1	Protein Coding	60	GC02M038066	2.13084650039673
P16152	CBR1	Carbonyl Reductase 1	Protein Coding	57	GC21P036069	0.671718597412109
P03372	ESR1	Estrogen Receptor 1	Protein Coding	67	GC06P201302	4.86715984344482
Q92731	ESR2	Estrogen Receptor 2	Protein Coding	61	GC14M064084	2.46142053604126
P23219	PTGS1	Prostaglandin-Endoperoxide Synthase 1	Protein Coding	61	GC09P122370	4.58845615386963
P27338	MAOB	Monoamine Oxidase B	Protein Coding	57	GC0XM043766	4.38460874557495
P0DMS8	ADORA3	Adenosine A3 Receptor	Protein Coding	55	GC01M111499	1.03919732570648
Q9UNQ0	ABCG2	ATP Binding Cassette Subfamily G Member 2 (JR Blood Group)	Protein Coding	62	GC04M088090	3.59786462783813
P42330	AKR1C3	Aldo-Keto Reductase Family 1 Member C3	Protein Coding	58	GC10P005035	1.83556735515594
P04054	PLA2G1B	Phospholipase A2 Group IB	Protein Coding	57	GC12M120322	1.12091851234436
P23141	CES1	Carboxylesterase 1	Protein Coding	61	GC16M055841	2.13710284233093
P37231	PPARG	Peroxisome Proliferator Activated Receptor Gamma	Protein Coding	65	GC03P012287	5.87569046020508
O00748	CES2	Carboxylesterase 2	Protein Coding	55	GC16P066934	0.546325206756592
P31639	SLC5A2	Solute Carrier Family 5 Member 2	Protein Coding	59	GC16P136877	0.888960599899292
P39900	MMP12	Matrix Metallopeptidase 12	Protein Coding	58	GC11M102862	1.03739082813263
P06746	POLB	DNA Polymerase Beta	Protein Coding	58	GC08P042338	2.41868567466736
P45452	MMP13	Matrix Metallopeptidase 13	Protein Coding	62	GC11M102942	3.065181017
P14555	PLA2G2A	Phospholipase A2 Group IIA	Protein Coding	60	GC01M019975	3.96829795837402
P39877	PLA2G5	Phospholipase A2 Group V	Protein Coding	51	GC01P020028	0.863021612167358
O15496	PLA2G10	Phospholipase A2 Group X	Protein Coding	55	GC16M014672	0.863021612167358
P56817	BACE1	Beta-Secretase 1	Protein Coding	59	GC11M117285	5.26774787902832
P36544	CHRNA7	Cholinergic Receptor Nicotinic Alpha 7 Subunit	Protein Coding	57	GC15P031923	0.194879993796349
P20151	KLK2	Kallikrein Related Peptidase 2	Protein Coding	58	GC19P050861	0.194879993796349
P19793	RXRA	Retinoid X Receptor Alpha	Protein Coding	61	GC09P134317	0.370410919189453
P05121	SERPINE1	Serpin Family E Member 1	Protein Coding	63	GC07P115646	3.47495174407959

Uniprot ID	Gene Symbol	Description	Category	Gifts	GC Id	Relevance score
P12931	SRC	SRC Proto-Oncogene, Non-Receptor Tyrosine Kinase	Protein Coding	63	GC20P037344	2.7270450592041
P00915	CA1	Carbonic Anhydrase 1	Protein Coding	60	GC08M085327	0.417015761137009
P10721	KIT	KIT Proto-Oncogene, Receptor Tyrosine Kinase	Protein Coding	66	GC04P054657	1.02214050292969
P35968	KDR	Kinase Insert Domain Receptor	Protein Coding	67	GC04M055078	3.61244368553162
P11362	FGFR1	Fibroblast Growth Factor Receptor 1	Protein Coding	67	GC08M038400	1.28003740310669
P08581	MET	MET Proto-Oncogene, Receptor Tyrosine Kinase	Protein Coding	67	GC07P116672	1.92105042934418
P07451	CA3	Carbonic Anhydrase 3	Protein Coding	54	GC08P085373	1.30871295928955
P16083	NQO2	N-Ribosyldihydronicotinamide: Quinone Dehydrogenase 2	Protein Coding	57	GC06P020569	2.76787281036377
P07858	CTSB	Cathepsin B	Protein Coding	63	GC08M011842	0.905553817749023
Q9NPH5	NOX4	NADPH Oxidase 4	Protein Coding	56	GC11M089324	7.4580340385437
P15121	AKR1B1	Aldo-Keto Reductase Family 1 Member B	Protein Coding	60	GC07M134442	4.92705535888672
P49759	CLK1	CDC Like Kinase 1	Protein Coding	48	GC02M200853	0.159118860960007
Q9Y463	DYRK1B	Dual Specificity Tyrosine Phosphorylation Regulated Kinase 1B	Protein Coding	58	GC19M039825	0.194879993796349
P06276	BCHE	Butyrylcholinesterase	Protein Coding	63	GC03M165772	3.05025315284729
Q16790	CA9	Carbonic Anhydrase 9	Protein Coding	60	GC09P035673	1.2693794965744
P11474	ESRRA	Estrogen Related Receptor Alpha	Protein Coding	59	GC11P064305	1.14602172374725
O95718	ESRRB	Estrogen Related Receptor Beta	Protein Coding	60	GC14P076310	1.00056874752045
P08069	IGF1R	Insulin Like Growth Factor 1 Receptor	Protein Coding	68	GC15P098648	3.36359763145447
P06213	INSR	Insulin Receptor	Protein Coding	66	GC19M007112	1.47538113594055
Q13627	DYRK1A	Dual Specificity Tyrosine Phosphorylation Regulated Kinase 1A	Protein Coding	63	GC21P037365	0.751031577587128
P17936	IGFBP3	Insulin-Like Growth Factor Binding Protein 3	Protein Coding	59	GC07M045912	3.10225105285645
P08253	MMP2	Matrix Metallopeptidase 2	Protein Coding	66	GC16P137330	5.8962140083313
Q8IXJ6	SIRT2	Sirtuin 2	Protein Coding	60	GC19M038878	2.89467620849609
P13726	F3	Coagulation Factor III, Tissue Factor	Protein Coding	58	GC01M095143	1.85605478286743
P43116	PTGER2	Prostaglandin E Receptor 2	Protein Coding	61	GC14P052314	0.874359130859375
Q07817	BCL2L1	BCL2 Like 1	Protein Coding	62	GC20M031664	6.46267938613892
P42338	PIK3CB	Phosphatidylinositol-4,5-Bisphosphate 3-Kinase Catalytic Subunit Beta	Protein Coding	61	GC03M138652	1.00288796424866
P11712	CYP2C9	Cytochrome P450 Family 2 Subfamily C Member 9	Protein Coding	60	GC10P094938	2.17815780639648
P08684	CYP3A4	Cytochrome P450 Family 3 Subfamily A Member 4	Protein Coding	62	GC07M107343	5.28683853149414
P42336	PIK3CA	Phosphatidylinositol-4,5-Bisphosphate 3-Kinase Catalytic Subunit Alpha	Protein Coding	64	GC03P179148	2.82230710983276
P55072	VCP	Valosin Containing Protein	Protein Coding	61	GC09M036512	0.251589000225067
P49763	PGF	Placental Growth Factor	Protein Coding	56	GC14M074941	3.09248757362366

Uniprot ID	Gene Symbol	Description	Category	Gifts	GC Id	Relevance score
P15692	VEGFA	Vascular Endothelial Growth Factor A	Protein Coding	61	GC06P043770	7.21721410751343
P25101	EDNRA	Endothelin Receptor Type A	Protein Coding	61	GC04P147480	0.803326010704041
O75460	ERN1	Endoplasmic Reticulum To Nucleus Signaling 1	Protein Coding	60	GC17M064039	2.02360963821411
P08254	MMP3	Matrix Metallopeptidase 3	Protein Coding	62	GC11M102835	3.66102027893066
P05067	APP	Amyloid Beta Precursor Protein	Protein Coding	63	GC21M025880	5.8942174911499
P49841	GSK3B	Glycogen Synthase Kinase 3 Beta	Protein Coding	64	GC03M119821	4.55795097351074
P10415	BCL2	BCL2 Apoptosis Regulator	Protein Coding	64	GC18M063123	10.8563852310181
P14780	MMP9	Matrix Metallopeptidase 9	Protein Coding	66	GC20P046008	6.33691167831421
P30291	WEE1	WEE1 G2 Checkpoint Kinase	Protein Coding	61	GC11P009573	0.761960566043854
P06239	LCK	LCK Proto-Oncogene, Src Family Tyrosine Kinase	Protein Coding	64	GC01P032251	0.894912719726563
P43405	SYK	Spleen Associated Tyrosine Kinase	Protein Coding	64	GC09P159630	2.05418539047241
P24864	CCNE1	Cyclin E1	Protein Coding	61	GC19P029811	0.764928221702576
P11802	CDK4	Cyclin Dependent Kinase 4	Protein Coding	66	GC12M061024	4.12770366668701
O14965	AURKA	Aurora Kinase A	Protein Coding	64	GC20M056370	0.523840188980103
P37840	SNCA	Synuclein Alpha	Protein Coding	64	GC04M089724	3.56944751739502
P18054	ALOX12	Arachidonate 12-Lipoxygenase, 12S Type	Protein Coding	57	GC17P174486	2.18446350097656
P41235	HNF4A	Hepatocyte Nuclear Factor 4 Alpha	Protein Coding	62	GC20P044355	0.555580973625183
P06493	CDK1	Cyclin Dependent Kinase 1	Protein Coding	60	GC10P060772	2.91745591163635
P25098	GRK2	G Protein-Coupled Receptor Kinase 2	Protein Coding	61	GC11P067266	0.159118860960007
Q00535	CDK5	Cyclin Dependent Kinase 5	Protein Coding	66	GC07M151053	0.63106369972229
P14635	CCNB1	Cyclin B1	Protein Coding	60	GC05P069167	3.6241147518158
P24941	CDK2	Cyclin Dependent Kinase 2	Protein Coding	63	GC12P055966	3.69670248031616
Q00526	CDK3	Cyclin Dependent Kinase 3	Protein Coding	48	GC17P176393	0.112514019012451

Supplementary Table 2. Mathematical model parameters of H_2O_2 and/or NGN-induced HT22 cells obtained using the Cole-Cole equation.

**Mathematical model parameters**	**Symbol/ unit**	**HT22**	**HT22 + H_2O_2**	**HT22 + H_2O_2 + NGN**
Cell volume fraction	Φ	0.25±0.03	0.26±0.03	0.26±0.03
The limiting impedance at high frequency	R_∞/Ω·m	-0.04±0.01	-0.03±0.00***	-0.03±0.00$^{\#}$
The first characteristic frequency	f_{C1}/Hz	(6.82±2.41)×10^5	(5.60±1.16)×10^{5*}	(9.68±0.47)×$10^{5\#\#\#}$
The first impedance increment	ΔR_1/Ω·m	0.45±0.07	0.48±0.13	0.40±0.13$^{\#}$
The first Cole-Cole parameter	β_1	0.75±0.04	0.74±0.02	0.86±0.040$^{\#\#\#}$
The second characteristic frequency	f_{C2}/Hz	(3.40±0.51)×10^7	(3.01±0.44)×10^{7**}	(2.51±0.70)×$10^{7\#\#}$
The second impedance increment	ΔR_2/Ω·m	0.32±0.07	0.33±0.06	0.36±0.01$^{\#\#}$
The second Cole-Cole parameter	β_2	0.70±0.04	0.70±0.03	0.66±0.07$^{\#\#}$
Residual	Res(Z')/%	1.04±0.28	1.19±0.17*	1.07±0.24
	Res(Z")/%	3.52±0.47	3.35±0.55	2.47±0.50$^{\#\#\#}$

Note: * denotes significance for the comparison between HT22 and HT22 + H_2O_2 groups; $^{\#}$ denotes significance for the comparison between HT22 + H_2O_2 and HT22 + H_2O_2 + NGN groups: $^{*/\#}p < 0.05$, $^{**/\#\#}p < 0.01$, $^{***/\#\#\#}p < 0.001$. For each concentration point within the tested range, data are presented as mean ± SD.

Supplementary Table 3. Molecular Dynamics Simulation Analysis of NGN–Target Protein Complexes.

**Complex System**	**Average RMSD (nm)**	**SD (nm)**	**Average Rg (nm)**	**SD (nm)**	**Average SASA (nm^2)**	**SD (nm^2)**
BCL2–NGN	0.212	0.013	1.490	0.008	89.5	1.66
ESR1–NGN	0.286	0.028	1.843	0.012	133.7	2.80
GSK3B–NGN	0.209	0.007	2.871	0.008	330.6	3.61
HO-1–NGN	0.173	0.012	2.376	0.009	206.3	2.46
MMP9–NGN	0.197	0.010	2.520	0.009	200.0	2.84
NRF2–NGN	0.304	0.022	1.413	0.024	79.9	2.72
PPARG–NGN	0.186	0.007	2.523	0.008	260.4	2.83
SRC–NGN	0.563	0.084	2.591	0.019	249.4	3.83

Notes: RMSD: root mean square deviation, reflecting complex stability during simulation. Rg: radius of gyration, indicating compactness of the complex. SASA: solvent-accessible surface area, related to the exposure of the complex to solvent.
